# A *Salmonella* nanoparticle mimic overcomes multidrug resistance in tumours

**DOI:** 10.1038/ncomms12225

**Published:** 2016-07-25

**Authors:** Regino Mercado-Lubo, Yuanwei Zhang, Liang Zhao, Kyle Rossi, Xiang Wu, Yekui Zou, Antonio Castillo, Jack Leonard, Rita Bortell, Dale L. Greiner, Leonard D. Shultz, Gang Han, Beth A. McCormick

**Affiliations:** 1 Department of Microbiology and Physiological Systems, 368 Plantation Street, Worcester, Massachusetts 01655, USA; 2 Department of Biochemistry & Molecular Pharmacology, 364 Plantation Street, Worcester, Massachusetts 01605, USA; 3 Program in Molecular Medicine, University of Massachusetts Medical School, 55 Lake Avenue North Worcester, Massachusetts 01655, USA; 4 The Jackson Laboratory, Bar Harbor, Maine 04609, USA

## Abstract

*Salmonella enterica* serotype Typhimurium is a food-borne pathogen that also selectively grows in tumours and functionally decreases P-glycoprotein (P-gp), a multidrug resistance transporter. Here we report that the *Salmonella* type III secretion effector, SipA, is responsible for P-gp modulation through a pathway involving caspase-3. Mimicking the ability of *Salmonella* to reverse multidrug resistance, we constructed a gold nanoparticle system packaged with a SipA corona, and found this bacterial mimic not only accumulates in tumours but also reduces P-gp at a SipA dose significantly lower than free SipA. Moreover, the *Salmonella* nanoparticle mimic suppresses tumour growth with a concomitant reduction in P-gp when used with an existing chemotherapeutic drug (that is, doxorubicin). On the basis of our finding that the SipA *Salmonella* effector is fundamental for functionally decreasing P-gp, we engineered a nanoparticle mimic that both overcomes multidrug resistance in cancer cells and increases tumour sensitivity to conventional chemotherapeutics.

## Abstract (web summary)

*S.* Typhimurium can grow selectively on tumours and decreases the cellular levels of the multidrug resistance transporter Pgp. Here, the authors reveal the SipA-dependent mechanism of Pgp down-regulation and produce a SipA-based gold nanoparticle that increases sensitivity to the anticancer drug doxorubicin.

## Graphical abstract

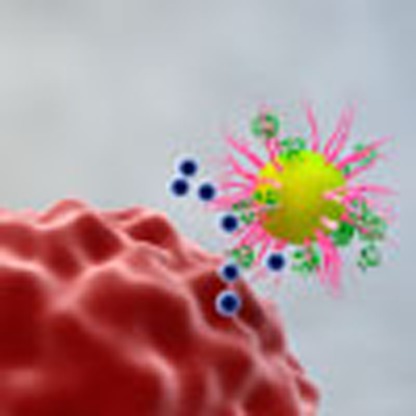

Bacteria have been investigated as therapeutic agents for tumours for over 150 years, when the German physicians W. Busch and F. Fehleisen first observed regression of tumours in cancer patients after accidental infections by erysipelas^1^. Later, the America physician William B. Coley injected *Streptococcus pyogenes,* as well as the heat-killed organism into patients with inoperable bone and soft-tissue sarcomas; these products became known as the Coley’s toxins^2^. Although the mechanisms underlying these observations were uncertain, it was known even then that bacteria exhibit immunostimulatory properties. Moreover, it has been known for over 60 years that anaerobic bacteria can selectively grow in tumours, and the conditions that permit anaerobic bacterial growth, such as impaired circulation and extensive necrosis, are found in many tumours signifying that bacterial therapeutic conduits may serve as a unique portal to a wide variety of malignancies^1^.

*Salmonella enterica* serovar Typhimurium (*S.* Typhimurium) is a facultative enteric pathogen that causes food poisoning in humans resulting in gastroenteritis. However, this pathogen can also selectively grow in tumours following systemic administration and is able to modulate numerous biochemical pathways across a broad spectrum of cell types (that is, gut, kidney, lung and macrophages)^3^. Therefore, the capacity to harness these traits affords unique opportunities to overcome many of the obstacles that hinder conventional chemotherapeutics. As an example, employing *Salmonella* as a potential monotherapy has been proposed in an emergent number of studies where this pathogen has been broadly developed as a delivery vector for cytokines, tumour antigens, and DNA-based vaccines^6^. Furthermore, recent evidence supports the use *S.* Typhimurium as an indirect activator of cytotoxic T cells against tumour antigens^7^. Yet, despite such therapeutic potential, the implementation of *Salmonella* as a viable treatment option has been unsuccessful clinically^8^, and remains compromised due to the risk of immune-mediated toxic responses at doses required for therapeutic efficacy^6^. There are also additional affects concerning genetic instability that could lead to possible failure of therapy or systemic infections^9^. Therefore, overcoming these principal limitations, particularly with respect to exploiting bacterial proteins in therapy, are key to advancing novel cancer treatment regimens.

*S.* Typhimurium initiates infection and controls the fate of the host cells by invading enterocytes predominantly located within the distal ileum, and has evolved the use of a needle-like structure, known as the type III secretion system to guide its pathogenesis^10^. By way of this sophisticated secretion system, numerous *Salmonella* effector proteins are secreted from the bacterium and then are translocated into the target cell cytosol. These secreted effectors function in the modulation of numerous signalling transduction pathways that are common targets in the development of therapeutics for inflammatory diseases and cancer^5^,^10^. Therefore, such secreted effectors have high potential as therapeutic agents, because they have co-evolved with the host and are extremely adept at interacting with host cell proteins.

We have recently uncovered a strong regulatory effect of the enteric pathogen, *S.* Typhimurium, on the multidrug resistance (MDR) transporter P-gp (P-glycoprotein). In particular, we found that colonization of human colon cancer cell lines by *S.* Typhimurium leads to a profound functional decrease and loss of protein expression in P-gp^11^. This was the first observation to link a microorganism that is targeted specifically to tumours with the regulation of MDR transporters. P-gp is encoded by *MDR1,* and is a MDR ATP-binding cassette membrane transporter responsible for one aspect of the (MDR) phenotype in cancer cells^12^. Recent reports have linked the overexpression of P-gp to adverse treatment outcomes in many cancers, thereby identifying this MDR phenotype as an important biologic target for pharmacologic modulation^12^,^13^. The nexus of this observation with reports documenting the ability of *S.* Typhimurium to target and selectively grow in tumours^4^ has led to the question of whether *Salmonella* or its products can be engineered to exploit MDR transporters for the development of new cancer therapeutics aimed at reversing drug resistance.

Herein, we reveal the *S.* Typhimurium type III secreted effector protein SipA as the key virulence factor responsible for modulating P-gp through a pathway involving caspase-3. Taking advantage of this property, we describe a new technology platform in which we construct a semi-synthetic ‘*Salmonella* nanoparticle mimic’ by engineering a gold (Au) nanoparticle scaffold loaded with a SipA corona that mimics the ability of *S.* Typhimurium to reverse MDR. Using this technology, we demonstrate that suppression of P-gp function can be achieved within a solid tumour to enhance efficacy and cytotoxicity of a non-targeted chemotherapeutic.

## Results

### SipA modulates the expression of P-gp

**Figure 1:**
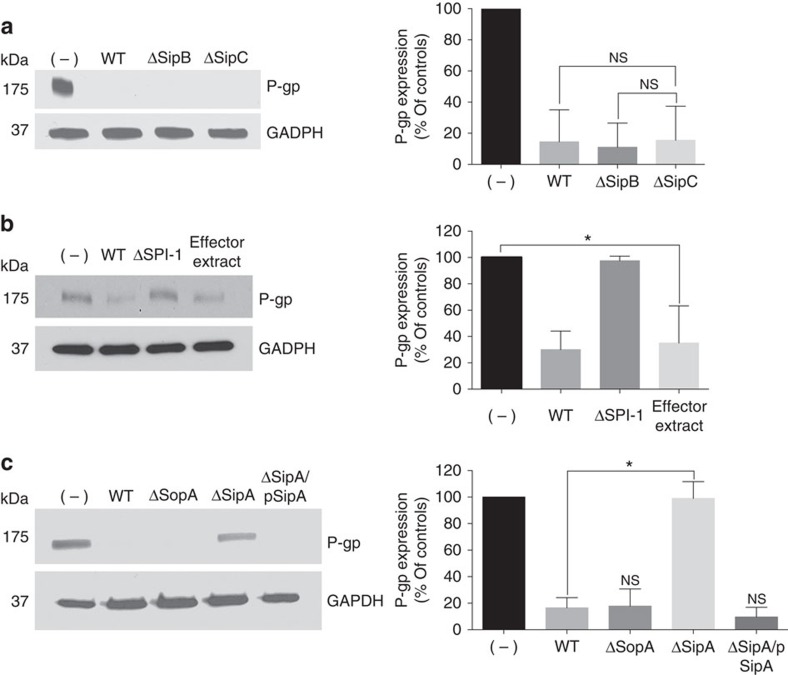
The *S*. Typhimurium effector protein SipA modulates the expression of P-gp by an extracellular effect. Western blot analysis showing P-gp expression in whole-cell lysates of HCT8 intestinal epithelial cell monolayers exposed to wild-type (WT) *S*. **Typhimurium SL1344, SL1344 type III secretion system mutants, or wild-type SL1344-derived secreted protein extracts. GAPDH probing served as a loading control. Densitometries were analysed by ImageJ and presented as relative to the untreated cells; data represent the mean± s.d. of three independent experiments. (**a**) HCT8 intestinal epithelial cell monolayers were left untreated (−) or infected with WT SL1344 or SL1344 type III secretion system translocon mutant strains (ΔsipB or ΔsipC) for 5 h. (NS, not significant (Student’s *t*-test). (**b**) HCT8 cells were infected with wild type SL1344 or an SL1344 SPI-1-deficient mutant strain, or exposed to WT SL1344-derived secreted protein extracts for 5 h, and then probed as in **a**. *P*=0.0164 (Student’s *t*-test); significantly different compared with negative controls. (**c**) HCT8 cells were infected with WT SL1344, SL1344ΔsopA or ΔsipA, or SL1344ΔSipA complemented with a vector expressing SipA (ΔSipA/pSipA) for 5 h, and then probed as in **a**. *P=0.0007 (Student’s *t*-test).

Since our prior study revealed that *S.* Typhimurium SPI-1 is necessary to modulate the expression of P-gp^11^, we began by screening *S.* Typhimurium type III secreted effectors to determine whether any are altered in their ability to modulate P-gp. We found that when HCT8 human intestinal carcinoma cell monolayers were exposed to *Salmonella* mutants of the type III secretion system translocon, ΔSipB or ΔSipC, these mutants maintained the ability to modulate P-gp (Fig. 1a). Phenotypically, the ΔSipB and ΔSipC translocon mutants are able to secrete effectors out through the type III secretion needle complex, but fail to translocate them into the host cells^14^. Our findings therefore suggest that a *Salmonella*-secreted effector could be modulating P-gp as a result of an extracellular interaction rather than due to its direct delivery into epithelial cells. To examine this possibility, HCT8 epithelial monolayers were exposed to an extract of secreted proteins that were isolated from *S.* Typhimurium (see Methods section). Since the treatment of this protein extract alone is sufficient to trigger the modulation of P-gp (Fig. 1b), we next examined individual *S.* Typhimurium type III secreted effector mutants to establish whether any fail to modulate P-gp. As shown in Fig. 1c, a *S.* Typhimurium ΔSipA mutant strain (EE633) is markedly reduced in its ability to modulate P-gp. The specificity of the Δ*sipA* mutant defect was verified by demonstrating that a plasmid, which expresses the *sipA* gene, restores the ability of the Δ*sipA* mutant to modulate P-gp to the approximate levels elicited by the wild-type strain (Fig. 1c).

Because normal intestinal epithelium display baseline expression of P-gp, we also assessed whether SipA could modulate the expression of P-gp in healthy murine intestinal epithelium *in vivo.* We evaluated the colonic expression of P-gp in BALB/c mice infected orally with 10^7^ CFU (colony-forming units) of either a *S.* Typhimurium strain that overexpresses SipA (AJK63), an isogenic ΔSipA mutant strain (EE633) or the parent wild-type *S.* Typhimurium (SL1344) strain. After 48 h of infection, the ΔSipA mutant failed to modulate the protein expression of P-gp as compared with the profound decrease in the expression of P-gp observed in the cohort of mice that were either infected with the SipA over-expressing strain (AJK63) or mice infected with the wild-type (Supplementary Fig. 1). Nevertheless, only modest differences in intestinal colonization between the ΔSipA strain (4.7 log_10_ CFU per mg of tissue±0.1 log_10_), as compared with either AJK63 (5.5 log_10_ CFU per mg of tissue±0.14 log_10_) or the wild-type (5.5 log_10_ CFU per mg of tissue±0.3) *S*. Typhimurium strain were noted (results are presented as mean±s.d. *n*=3).

**Figure 2:**
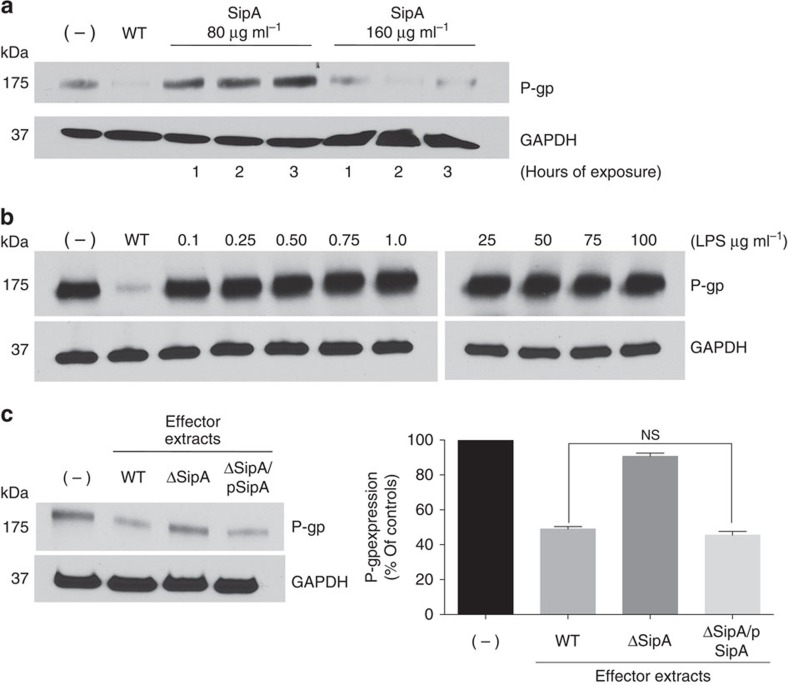
SipA downregulates P-gp expression in a dose-dependent manner. (**a**) HCT-8 cell monolayers were left untreated (−), or infected with wild-type SL1344, or exposed to 80 μg ml^−1^, or 160 μg ml^−1^ of purified SipA over a time course of 3 h. Normalized whole-cell lysates were then probed with P-gp and GAPDH. (**b**) HCT8 cell monolayers were infected with wild type SL1344 or exposed to purified lipopolysaccharide (LPS) from *S*. Typhimurium (0.1 to 100 μg ml^−1^) for 3 h, and then probed as in **a**. (**c**) HCT8 cell monolayers were exposed to secreted protein extracts from SL1344 wild-type, ΔsipA or ΔSipA/pSipA for 3 h, and then probed as in **a**. Densitometry was analysed by ImageJ and presented as relative to the untreated cells; data represent the mean±s.d. of three independent experiments. (NS, not significant (Student’s *t*-test). All western blots were performed at least three times independently and the results shown are from one representative experiment.

We next examined whether SipA alone could induce the modulation of P-gp without the influence of *Salmonella* or its type III effectors. Thus, affinity-purified hemagglutinin tagged SipA (purified-SipA) was added to PBS buffer overlying washed HCT8 cells. Exposure of cell monolayers to 80 or 160 μg ml^−1^ of purified-SipA over a period of 3 h resulted in a dose-dependent ability to modulate P-gp to the same degree as wild-type *S.* Typhimurium (Fig. 2a); this affect was not attributed to trace the amounts of lipopolysaccharide (Fig. 2b). Moreover, to further validate that SipA was responsible for the modulation of P-gp, HCT8 cell monolayers were exposed to an extract of secreted proteins isolated from the *S.* Typhimurium ΔSipA mutant (see Methods section). This extract contained all *S.* Typhimurium secreted effectors with the exception of SipA, and as shown in Fig. 2c, failed to modulate P-gp. Cell monolayers were also exposed to a secreted protein extract from the complemented mutant (*S.* Typhimurium ΔSipA/pSipA), which was rescued in its ability to modulate P-gp (Fig. 2c).

### Mechanism of SipA action on P-gp

**Figure 3 f3:**
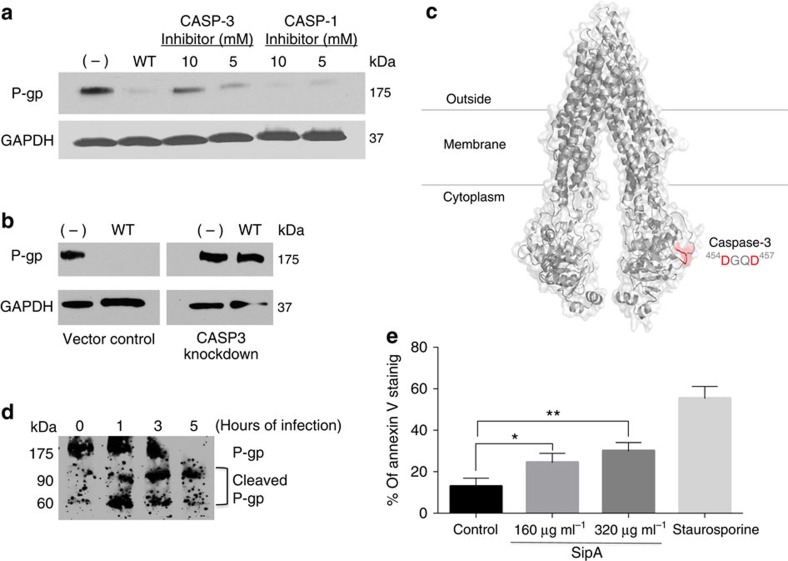
*S*. Typhimurium modulates P-gp expression through a caspase-3-dependent mechanism. (**a**) HCT8 cell monolayers were left untreated (−) or infected with wild-type SL1344 in the presence or absence of pharmacological inhibitors of CASP-3 or CASP-1 (negative control) for 5 h. Normalized whole-cell lysates were then probed with P-gp and GAPDH. The data represent a western blot analysis from an individual experiment performed at least three times. (**b**) HCT8 cell monolayers transfected with a nonspecific siRNA vector control or with siRNA aimed at decreasing CASP-3 expression were left untreated or infected with wild-type SL1344. Whole-cell lysates were then probed as in **a**. (**c**) Three-dimensional structure of mouse P-gp (PDB ID, 3G5U) depicted as cartoon and transparent surface. The cytoplasmic Caspase-3 cleavage site (^454^DGQD^457^) is shown in red. The putative CASP3 site ^164^DVHD^167^ is not shown. Numbers refer to the position of the amino acids in the protein sequence. (**d**) HCT8 cell monolayers were infected with wild type SL1344 for 1, 3 or 5 h, and then probed using a P-gp antibody capable of detecting P-gp cleavage products. Progressive P-gp modulation was accompanied by the occurrence of 90- and 60-kDa cleavage products. (**e**) Anexin-V staining was used as measurement of apoptosis in HCT8 cells 3 h post incubation with 160 or 320 μg ml^−1^ of purified SipA. Staurosporine treatment served as the positive control. Shown is the average of three independent experiments with error bars indicating s.d.; *P=0.0076, **P=0.0009 (Student’s *t*-test).

We have previously shown that protein expression of P-gp is modulated in *Salmonella*-infected epithelial cells without a corresponding decrease in P-gp messenger RNA. This observation is consistent with a mechanism of P-gp protein cleavage and/or rearrangement from the cell membrane rather than the regulation of gene expression. Evidence that cells are able to functionally modulate P-gp through a mechanism involving protein cleavage/degradation involving caspase-3 has been recently documented in human T-lymphoblastoid CEM cells^15^. Moreover, we have previously shown that the SipA effector protein is necessary and sufficient to promote the activation of caspase-3 (CASP3)^16^ most likely through a pathway involving the tetraspanning membrane protein PERP^17^. Thus, we examined the protein expression of P-gp in HCT8 cells following infection from *S.* Typhimurium in the absence and presence of a pharmacological inhibitor of CASP3. As shown in Fig. 3a, western blot analysis demonstrates that CASP3, but not CASP1 inhibition (which was used as the negative control), prevented *S.* Typhimurium from downregulating P-gp. A similar outcome was also observed using HCT8 cells knocked down for the expression of CASP3 (Fig. 3b).

Since *in silico* modelling of mouse P-gp (which is 89% identical to the human P-gp) revealed two surface-exposed CASP3 cleavage motifs (DQGD and DVHD; Fig. 3c), we next performed a P-gp degradation protocol (see Methods section) and found that HCT-8 cells infected with *S.* Typhimurium showed a progressive reduction in the expression of P-gp; this correlated with the appearance of the predicted CASP3 cleavage products of P-gp (90 kDa and ∼60 kDa), as calculated from the *in silico* model (Fig. 3c,d). The lower 25-kDa band was not resolved most likely due to lack of antibody recognition. Taken together, these observations suggest that the ability of *S.* Typhimurium to degrade P-gp via SipA depends on its ability to activate CASP3, and is consistent with our previous findings showing that SipA activates CASP3 (refs 16). Since CASP3 is a frequently activated death protease it is perhaps not surprising that we also observed SipA to be an inducer of apoptosis, as shown for the HCT8 colonic carcinoma cell line (Fig. 3e).

**Figure 4:**
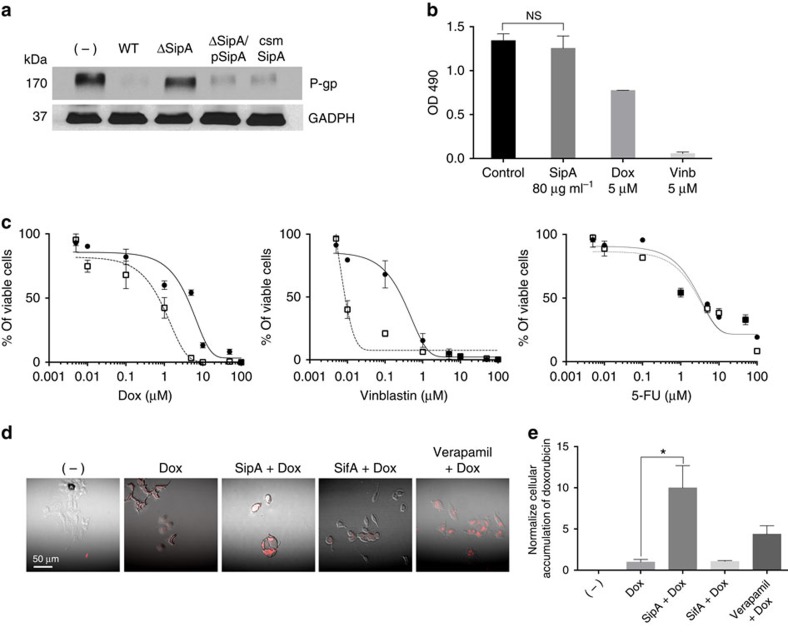
SipA enhances the cytotoxic effect of doxorubicin on HCT8 cells. (**a**) Alteration of the SipA CASP-3 motif did not alter the ability of *S*. Typhimurium to modulate P-gp. HCT8 cells were infected with wild-type SL1344, SL1344 ΔsipA or SL1344 ΔSipA complemented with a vector expressing SipA with a mutated CASP-3 cleavage motif (ΔSipA/pCSM-SipA) for 5 h. Whole-cell lysates were normalized for protein levels and probed for P-gp. GAPDH served as a loading control. The data represent a western blot analysis from an individual experiment performed at least three times. (**b**) HCT8 cells were treated with purified SipA (80 μg ml^−1^), doxorubicin (5 μM) or vinblastine (5 μM) for 72 h. Cytotoxicity was measured using a colorimetric cell proliferation assay (see Methods section). (NS, not significant (Student’s *t*-test). Data were obtained from three independent experiments performed in triplicate and error bars indicate s.d. (**c**) Dose–response curves of doxorubicin and vinblastine (P-gp substrates), as well as 5-FU (not a P-gp substrate) in the absence (circle) or in the presence of purified-SipA (square). HCT8 cells were grown for 72 h with specific concentrations of the cytotoxic drugs (in μM) with or without purified-SipA (80 μg ml^−1^). Cells viability was measure by CellTiter96 Aqueous One solution cell proliferation assay (see Methods section). Dose–response curves were derived from three independent experiments; error bars indicate±s.d. (*n*=3). Absent error bars indicate that error fell within the symbol. (**d**) Purified-SipA increases the intracellular accumulation of doxorubicin. HCT8 cells were treated with media only, doxorubicin, doxorubicin combined with purified-SipA, doxorubicin combined with purified-SifA or doxorubicin combined with verapamil. Accumulation of doxorubicin was evaluated by confocal microscopy as described in the Methods section. Red colour intensity represents intracellular doxorubicin accumulation. (**e**) The level of doxorubicin staining was quantified using the ImageJ software. Data represent the average of five readouts and error bars indicate s.d.; *P=0.001 (Student’s *t*-test).

Because SipA contains a CASP3 cleavage recognition site that also has the potential to be cleaved for functional processing^16^, we next examined whether the ability of SipA to modulate P-gp expression requires CASP3 cleavage. We created an isogenic SipA strain (ΔSipA/pCSM-SipA)^16^, which harbours a point mutation in the caspase-3 recognition motif, rendering this effector insensitive to CASP3 cleavage, and found this strain still retains the ability to effectively reduce P-gp protein expression in HCT8 cells (Fig. 4a). As these results indicate that SipA, itself, does not depend on CASP3 cleavage to modulate P-gp, our future studies will be directed at probing the precise domain on SipA responsible for such P-gp regulation.

### SipA potentiates the effect of cytotoxic drugs in vitro

Since P-gp is as an important biological target for pharmacological modulation^12^,^13^, and our data strongly infer that SipA promotes P-gp degradation, we next evaluated whether SipA could improve the cytotoxic activity of known chemotherapeutic drugs (doxorubicin and vinblastine), which are also P-gp substrates. We used a well-established colorimetric cell proliferation assay (see Methods section) in which HCT8 cells were cultured in media with or without purified-SipA along with doxorubicin or vinblastine (ranging in concentration from 0.001 to 100 μM). Although the concentration of purified-SipA (80 μg ml^−1^) was not toxic to the cells (Fig. 4b), exposure of purified-SipA to either doxorubicin or vinblastine induced a profound shift to the left in the drug response curve (Fig. 4c). Specifically, the calculated drug concentration required for 50% cell mortality (IC_50_) shifted from 5.05±0.71 to 0.85±0.14 μM for doxorubicin and from 0.35±0.24 to 0.037±0.008 μM for vinblastin. Conversely, purified SipA did not change the IC_50_ of 5-fluorouracil, a cytotoxic drug not effluxed by P-gp (Fig. 4c). Similarly, we observed that SipA could prevent the extrusion of cytotoxic compounds, such as doxorubicin, from HCT8 colon cancer cells (Fig. 4d). Moreover, as shown in Fig. 4e, quantitative analysis of the intracellular accumulation of doxorubicin in cells that were incubated with SipA was as high, if not higher, as compared with verapamil, a well-known competitive P-gp inhibitor. An irrelevant *S.* Typhimurium secreted effector SiFA served as the negative control.

### Construction of *Salmonella* nanoparticle mimic

Capitalizing on the ability of SipA to degrade P-gp and increase drug cytotoxicity, we next sought to build a *Salmonella* nanoparticle mimic made by fusing a nanoparticle with a SipA corona to be applied as an effective chemotherapeutic adjuvant. For this purpose, we selected gold nanoparticles as a scaffold because these particles are inert^18^,^19^, easily synthesized and modified, and stabilize conjugated pharmaceutics (for example, proteins^20^ and small-molecule drugs^18^). We fabricated 15-nm gold nanoparticles since nanoparticles that are <100 nm have a unique enhanced permeation and retention effect, and can, therefore effectively extravasate and remain within interstitial spaces, resulting in a much higher payload concentration of SipA at tumour sites^21^,^22^,^23^,^24^,^25^,^26^,^27^,^28^,^29^,^30^,^31^,^32^,^33^.

**Figure 5:**
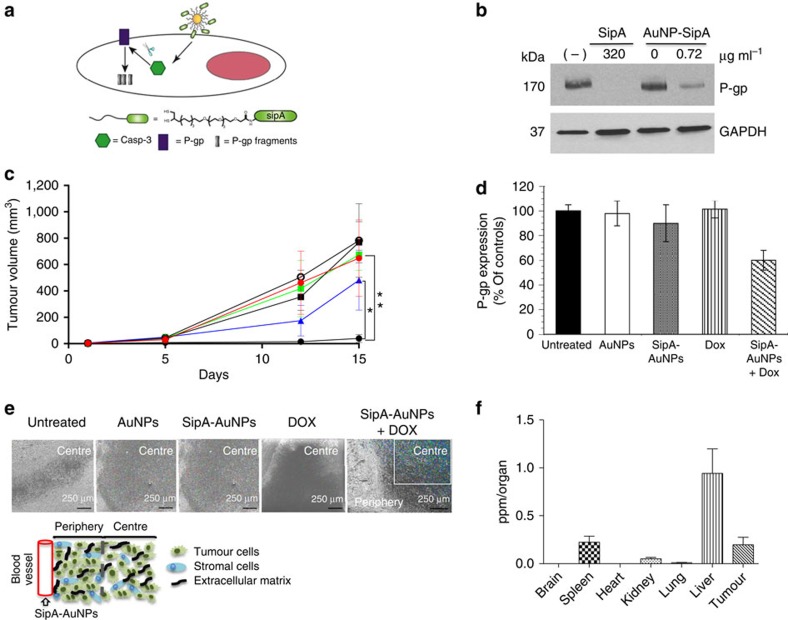
The combined effect of SipA-AuNP delivery and exogenous doxorubicin treatment prevents tumour growth in a murine model of colon cancer. (**a**) Schematic presentation of P-gp knockdown mechanism via SipA-AuNP. (**b**) SipA-AuNPs decrease the expression of P-gp at a SipA dose >100 times lower than free SipA. HCT8 cell monolayers were left untreated (−), exposed to 320 μg ml^−1^ of purified SipA, AuNP alone or AuNP-SipA (0.72 μg ml^−1^ of SipA) for 3 h. Whole-cell lysates were normalized for protein levels and probed for P-gp. GAPDH served as the loading control. All experiments were performed at least three times and the results shown are from one representative experiment. (**c**) The SipA-AuNPs conjugates improve the efficacy of doxorubicin. Balb/c mice bearing subcutaneous CT26 tumours (mean tumour volumes of ∼0.5 mm^3^) received i.p. treatments for 15 days (described in the Methods section) with AuNP alone (black square), SipA-AuNPs (green square), doxorubicin (blue triangle), AuNP in combination with doxorubicin (DOX) (black circle), SipA-AuNP in combination with DOX (black dots) or left untreated (red dot). Values indicate mean±s.d. (*n*=6); *P=0.0008, **P<0.0001 (one-way ANOVA). (**d**) P-gp expression in the tumours shown in **c** was evaluated by western blot. Tumours were homogenized and lysed, whole-cell lysates were normalized for protein levels and probed for P-gp. Levels of P-gp were quantified by densitometry and presented on the bar chart. Densitometry was performed using ImageJ and results are presented as relative to the untreated cells and error bars indicate s.d. (**e**) Accumulation of gold nanoparticles in the tumours shown in **c** was evaluated by SEM and X-ray microanalysis. Colour intensity represents tumour penetration. The sections of tumour were imaged for X-ray analysis and X-ray mapping as described in the Methods section. The cartoon inset depicts a schematic representation of the tumour architecture and basic components of cancer tissues. (**f**) BALB/c mice bearing subcutaneous CT26 tumours (mean tumour volumes of approximately 0.5 mm^3^) received i.p. treatments for 15 days as described in the Methods section. At necropsy, brain, spleen, heart, kidney, lung, liver and tumour tissues were collected. Tissue accumulation of SipA-AuNPs was determined by elemental analysis. Data represent the mean accumulation±s.d. (*n*=6). ANOVA, analysis of variance.

Although substantial progress has been made in promoting the use of AuNPs for genetic material and as small molecular drug-delivery systems, the delivery of functional proteins with retention or enhanced activity has been challenging due to inadequate maintenance of protein recognition and structure retention. To overcome this limitation, we designed surface ligands for direct conjugation of SipA to the AuNP by inserting biocompatible tetra(ethylene glycol) (TEG) spacers (see Methods section, Fig. 5a). This adaptation reduces non-specific interactions and absorption, and provides additional degrees of freedom and polyvalency for enhancing the conjugated protein’s activity. Moreover, the carboxylate terminus creates a platform for subsequent SipA coupling. Last, we covalently attached SipA to the carboxyl modified AuNP to avert protein dissociation or aggregation (see Methods section, Fig. 5a).

To determine the ratio of AuNP to conjugated SipA proteins, we next exposed the SipA-AuNP to sodium cyanide, which decomposes the gold particle core. The remaining SipA in solution was then dialysed for 2 days, and thereafter, concentrated, trypsin digested and measured with an Agilent Q-TOF 6538 mass spectrometer coupled with an Agilent HPLC 1200. Peptide IPEPAAGPVPDGGK ([M+2H]^2+^, *m*/*z* 652.8505) from the SipA protein was identified through MS/MS spectral match, and chosen as a surrogate for protein quantification. On the basis of mass spectrometry analysis, the binding ratio of AuNP:SipA is 1: 6 (Supplementary Fig. 2).

Subsequent *in vitro* testing of the SipA-conjugated AuNP with colonic tumour cells revealed that the design of this novel nanoparticle profoundly increases the stability of surface-bound SipA protein and reduces P-gp expression in cancer cells at SipA doses that are >100 times lower than in free unbound SipA (Fig. 5b). Such enhanced SipA functionality is most likely due to the large surface (the volume ratio of AuNP), which markedly stabilizes SipA proteins by preventing the conjugated proteins from degradation. In addition, the polyvalency of SipA proteins on the surface of single AuNP may afford a potentiation effect, which does not exist in free-bound SipA.

### *Salmonella* nanoparticle mimic improves efficacy *in vivo*

We next sought to determine whether the design of the *Salmonella* nanoparticle mimic improves the efficacy of doxorubicin, a known chemotherapeutic drug, *in vivo*. We used a well-established subcutaneous murine colon cancer model as a prototypical model to study cancers that are known to overexpress P-gp^34^. Disease in this model is induced by the subcutaneous injection of CT26 colon cancer cells in ∼8-week-old BALB/c mice. The formation of palpable tumours (∼0.5 mm^3^ in size) denotes day 1 of the experiment. Mice were then intraperitonially (i.p.) injected with 2.5 pmols per day of SipA-AuNP (containing 15 pmoles or 1.1 μg SipA) for 2 days before i.p. treatment of a single dose of doxorubicin (10 mg kg^−1^). Since the key objective is to assess whether the SipA-AuNP itself is able to improve doxorubicin efficacy, 10 mg kg^−1^ identifies a concentration of the drug that we determined displays a minimal effect on tumour size. This treatment was followed by SipA-AuNP i.p. injections every 48 h for 15 days, after which, doxorubicin efficacy was assessed by the tumour volume in mm^3^. As shown in Fig. 5c, the tumour volume following the combined treatment (SipA-AuNP bacterial mimic+doxorubicin) was significantly less than the tumour volumes following either SipA-AuNP or doxorubicin treatment performed alone. It is worth noting that the expression of P-gp in tumours in which mice received only the SipA-AuNP treatment was reduced modestly (∼10%). It is likely that the different microenvironments encountered by the stable dose of SipA-AuNP accounts for these findings. For example, in the SipA-AuNP and doxorubicin combination treatment group, the potentiation effects of P-gp inhibition coupled with the chemotherapeutic drug were marked by profound decreases in both the tumour size and the number of cells, which enabled the SipA-AuNP to further penetrate the tumour and act on cells at an effective concentration. Yet, in contrast, the cohort receiving only the SipA-AuNP regimen, encountered tumours with a high cell proliferation rate, which might have diluted out the effect of the SipA-AuNPs.

On examining the toxicity and biodistribution, we further noted that mice treated with the *Salmonella* nanoparticle mimic did not exhibit signs of toxicity as monitored through their behaviour, such as fatigue, loss of appetite (weight loss), or changes in fur texture. Liver enzymes and blood chemistries were also found within normal limits throughout the duration of the experiment (Supplementary Fig. 3). In addition, no significant histological changes were detected in major organs (that is, brain, heart, kidney, ling and liver; Supplementary Fig. 4). However, P-gp expression is significantly diminished in tumours in which mice received the SipA-AuNP and doxorubicin combination regimen (Fig. 5d). This is consistent with our observation that Au was found at higher density in tumours of the mice that were treated with the SipA-AuNP/doxorubicin combination therapy (Fig. 5e), and were preferentially located in the center of the tumour. By contrast, in mice treated with either the SipA-AuNP or the AuNP alone, the tumours exhibited a diffuse distribution of the Au. Such findings were also confirmed by biodistribution assessment of the *Salmonella* nanoparticle mimic, in which accumulation of Au within the tumour in addition to major organs, was measured by elemental analysis (Fig. 5f; Elemental Analysis Inc., Lexington KY). Collectively, these observations reveal considerable penetration of the *Salmonella* nanoparticle mimic in the subcutaneous tumour, highlighting the ability of the bacterial nanoparticle mimic to effectively accumulate in tumours.

### Affects of SipA are broad spectrum

**Figure 6:**
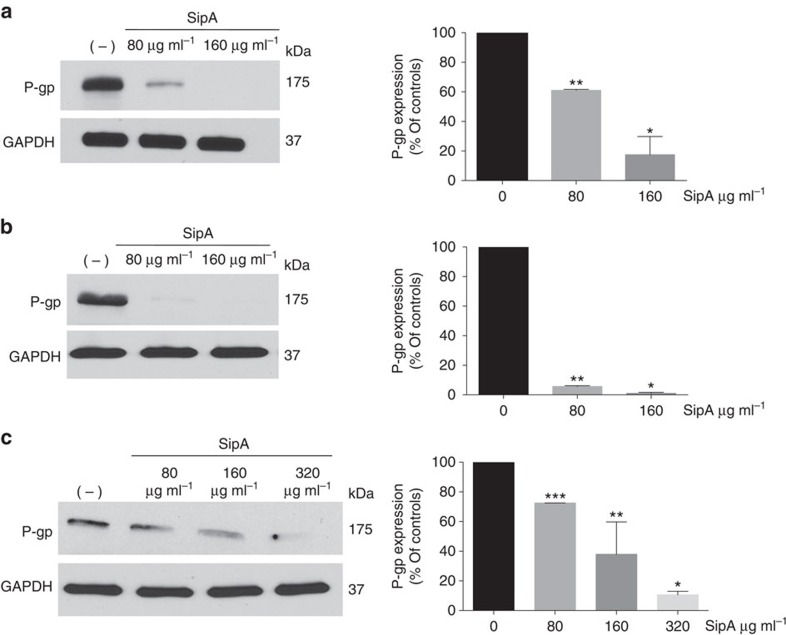
SipA-induced P-gp downregulation is conserved in other cancer cell types. (**a**) MCF-7 breast adenocarcinoma cells were left untreated (−), or exposed to 80 μg ml^−1^, or 160 μg ml^−1^ of purified-SipA for 3 h. Normalized whole-cell lysates were then probed for P-gp and GAPDH. Densitometry was analysed by ImageJ and presented as relative to the untreated cells; data show the mean of three independent experiments±s.d. *P=0.0003; **P<0.0001 (Student’s *t*-test), significantly different compared with negative controls. (**b**) UM-UC-3 human bladder carcinoma cells were left untreated, or exposed to 80 μg ml^−1^, or 160 μg ml^−1^ of purified-SipA for 3 h, and then probed and analysed as in **a**; data show the mean of three independent experiments±s.d.*P<0.0001; **P<0.0001 (Student’s *t*-test), significantly different compared with negative controls. (**c**) SU10 diffuse large B-cell lymphoma cells were left untreated or exposed to 80, 160 or 320 μg ml^−1^ of purified-SipA for 3 h, and then probed and analysed as in **a**; data show the mean of three independent experiments±s.d. *P<0.0001; **P=0.0078;***P<0.0001 (Student’s *t*-test), significantly different compared with negative controls (Student’s *t*-test). All experiments were performed at least three times and the results shown are from one representative experiment.

Because P-gp expression is documented to be upregulated in several types of malignancies, and contributes to their poor prognosis^12^,^13^, we assessed whether the ability of SipA to modulate P-gp is broad spectrum. Similar to colonic cancer cell lines, purified-SipA was exposed to cell monolayers of different cancer cell types of epithelial origin that are also known to overexpress P-gp, such as MCF-7 (breast adenocarcinoma) and UM-UC-3 (human bladder carcinoma). Compared with the buffer control, the exposure of purified-SipA to MCF-7 and UM-UC-3 cells also reduced the expression of P-gp in a dose-dependent manner demonstrating 82% and 99% reduction, respectively (Fig. 6). Analogous to the colon carcinoma cell lines, SipA markedly enhanced the cytotoxic activity of P-gp substrates, such doxorubicin and vinblastine in breast adenocarcinoma (MCF-7) and human bladder carcinoma (UM-UC-3) *in vitro* (Supplementary Fig. 5). The ability of SipA to modulate P-gp is additionally not restricted to tumour cells of epithelial origin given that SipA modulates the expression of P-gp in tumour cells originating from lymphoid tissues, as well (Fig. 6c).

**Figure 7:**
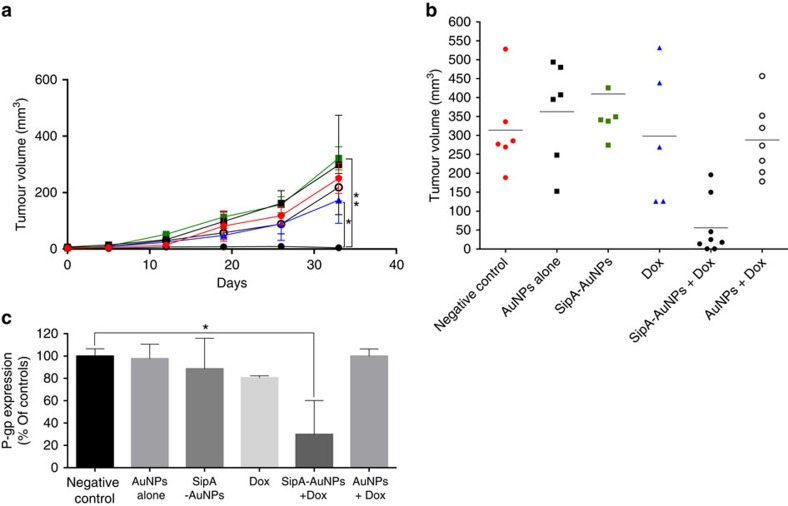
SipA-AuNP combined exogenous doxorubicin treatment prevents tumour growth in a NRG humanized model of primary breast cancer. (**a**) Primary breast cancer tumour growth curves after 33 days of i.p. treatments (described in the Methods section) with AuNP alone (black square), SipA-AuNPs (green square), doxorubicin (blue triangle), AuNP in combination with DOX (black circle), SipA-AuNP in combination with DOX (black dot) or left untreated (red dot). Results represent mean±s.d. (*n*=3) tumour volume mm^3^; *P=0.0241, **P=0.0002 (one-way ANOVA). (**b**) Primary breast cancer tumours volumes at day 33 after i.p. treatments as mentioned above. Results represent mean±s.d.; (*n* of at least five mice per group). (**c**) P-gp expression in the tumours shown in **a** was evaluated by western blot. Tumours were homogenized and lysed, and whole-cell lysates were normalized for protein levels and probed for P-gp. Levels of P-gp were quantified by densitometry using ImageJ. Results are presented as relative to the untreated cells. Data represent means±s.d. *P=0.0171 (Student’s *t*-test). ANOVA, analysis of variance.

Because breast cancers also express high levels of P-gp, and since we have validated that SipA enhances the cytotoxic activity of breast adenocarcinoma cells (above), we next assessed the broad-spectrum capabilities of the SipA nanoparticle mimic in a humanized mouse model^36^,^37^ of primary human breast cancer. Disease in this model is induced by xenograft implantation of human primary breast tumours into the mammary fat pad of ∼8-week-old immunodeficient NOD-*Rag1*^*null*^ *IL2rg*^*null*^ (NRG) mice. We employed a similar treatment strategy as above for the colon carcinoma model, but because NRG mice exhibit an elevated sensitivity to doxorubicin (as compared with the murine subcutaneous colon cancer model) the dose of the drug was reduced to 2 mg kg^−1^ every 2 weeks (see Methods section). This treatment was followed by SipA-AuNP i.p. injections every 48 h for 33 days, after which, doxorubicin efficacy was assessed by the tumour volume in mm^3^. As shown in Fig. 7, assessment over 33 days showed that the tumour volume following the combined treatment (SipA-AuNP bacterial mimic+doxorubicin) was profoundly less than the tumour volumes of either SipA-AuNP or doxorubicin treatment alone (Fig. 7a,b). In addition, a 70% reduction in P-gp protein expression was observed in tumours in which mice received the SipA-AuNP and doxorubicin combination regimen, as compared with untreated controls (Fig. 7c). Liver enzymes and indicators of renal health were found within normal limits throughout the duration of the experiment (Supplementary Fig. 6).

## Discussion

P-gp belongs to the ATP-binding cassette family and functions as a transmembrane efflux pump that translocates its substrates from an intracellular to an extracellular domain. Together with xenobiotic-metabolizing enzymes, constitutive P-gp expression in normal healthy tissues is believed to be an important protective mechanism against potentially toxic xenobiotics. However, during disease states, such as cancer, P-gp is recognized as a major barrier to the effective cytotoxic effect of systemically administered anti-neoplastic drugs, and as a consequence, resistance to chemotherapy remains an obstacle to the successful treatment of certain cancers. Therefore, formulating novel P-gp modulators as a way to revert MDR in human cancers remains a principal area of investigation^38^.

While advances in cell biology have strengthened the fundamental understanding of how malignant cells survive toxic insults and become resistant to antineoplastics, these investigations also yield evidence that P-gp’s substrate specificity and mechanism of export is more sophisticated than first realized; this efflux transporter contributes to anti-neoplastic resistance by active drug extrusion, and elevation of the cellular apoptotic threshold. Efforts to effect a more durable suppression of P-gp function have focused on the downregulation of MDR1 (the gene encoding P-gp) expression through various RNA interference strategies^38^,^39^,^40^, such as hammerhead ribozymes, RNA antisense, and siRNA. While effective *in vitro*, these genetic methods have been met with complications in *in vivo* delivery approaches and accompanied off-target effects, which, to date, has hindered their clinical translation^41^. Despite >20 years of effort, a large number of clinical trials with Pgp-modulating agents have been conducted, so far with poor success, as these drugs were either vastly ineffective or only effective at toxic doses^38^.

We have recently uncovered a strong regulatory effect of the enteric pathogen, *S.* Typhimurium, on the MDR transporter Pgp. In particular, we found that colonization of human colon cancer cell lines by *S.* Typhimurium leads to a profound functional decrease and loss of protein expression in P-gp. This was the first observation to link a microorganism that is targeted specifically to tumours with the regulation of MDR transporters.

In the current report, we now identify the *S.* Typhimurium type III secreted effector protein SipA as the key virulence factor responsible for functionally downregulating Pgp, and further exploit this virulence determinant in the development of a new strategy aimed at reversing MDR.

The main reason for failure of MDR-reversing agents is thought to be the pharmacokinetic interaction with the anticancer agents^40^. Typically, a poor bioavailability profile results from high metabolism and high serum protein binding, which considerably reduces the concentration of drug available for action and increases the demand for higher doses to be administered. A key biochemical feature that prompted the concept of the *Salmonella* nanoparticle mimic was the finding that SipA could markedly improve the efficacy of commonly used chemotherapeutic drugs, such as doxorubicin and vinblastine through a mechanism that apparently involves CASP3 cleavage, which degrades P-gp. Assessment of the P-gp cleavage products requires the P-gp mAb C494 clone^15^, which binds to a common epitope in both the third and the sixth extracellular loops of P-gp, allowing detection of the two cleavage products. Evidence this model has been documented in human T-lymphoblastoid CEM cells^15^ and is consistent with our observation that SipA robustly induces the intracellular accumulation of doxorubicin in colonic cancer cells.

We therefore have taken the first steps in engineering a novel SipA-AuNP conjugate to combat MDR by engineering a SipA conjugated nanoparticle system that capitalizes on the unique chemical and physical properties of Au-NPs, the biochemical functional activities of SipA, and pharmaceutical effectiveness of an FDA approved chemotherapeutic agent, such as doxorubicin. Although substantial progress has been made in promoting the use of AuNPs for genetic material and small molecular drug-delivery systems, the delivery of functional proteins with retention or enhanced activity remains difficult due to the complexity of protein recognition and structure retention on nanoparticle surface. To circumvent this problem, we inserted a biocompatible TEG spacer between the SipA protein and the surface of the AuNP. Applying this design, we found that AuNP-conjugated SipA can reduce P-gp expression at a SipA dose >100 times lower than free unbound SipA. Such enhanced SipA functionality is most likely due to the large surface:volume ratio of nanoparticle, which markedly stabilizes SipA proteins by preventing the conjugated proteins from degradation. In addition, polyvalency of SipA proteins on the surface of single AuNP may offer a unique potentiation effect, which does not exist in free-bound SipA.

To demonstrate proof of principle of this semi-synthetic *Salmonella* nanoparticle mimic, we employed a colorectal carcinoma model since this type of cancer is known to be one of the tumours with the highest expression of the *MDR1* gene (encoding for P-gp)^42^ and in addition, acquires high expression of P-gp in the course of carcinogenesis. This well-established subcutaneous murine colon cancer model also complements the full spectrum of tumour development with generation of viable tumours within 2 weeks after implantation, and exhibits a low rate of metastasis. Although this model fails to replicate the original anatomical site, as compared with orthotopic (*in situ*) models, such features are sufficient to assess key functional parameters of tumourigenesis in response to our drug formulation. In addition, proof of principle was further demonstrated using a humanized mouse model of primary breast cancer. This model is based on the inability of the immunodeficient NRG mice to generate mature T and B cells, as well as natural killer cells, and thus enable the adequate engraftment of tissues of human origin. Furthermore, reports suggesting the induction of P-gp over-expression in breast cancers following treatment with P-gp substrate-containing regimens, make breast cancer cells an ideal candidate for our studies^43^.

**Figure 8:**
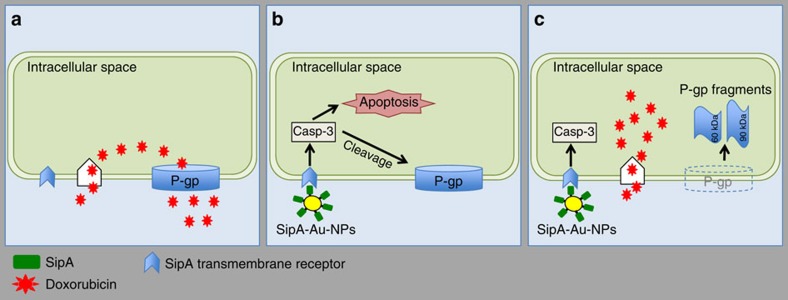
Working model of SipA downregulation of P-gp. (**a**) Cancer cells express different types of ABC transporters, especially P-gp, to gain multidrug resistance. This allows tumour cells to extrude cytotoxic drugs from the intracellular space. (**b**) The SipA-AuNP may act extracellularly, by interacting with a transmembrane receptor to induce a CASP3 dependent cleavage of P-gp. The activation of caspase-3 also results in apoptosis; a cell death process. (**c**) Cleavage of P-gp results in the appearance of two protein fragments of about 90 and 60 kDa. Such cleavage destroys the P-go scaffold essentially removing this transporter from the plasma membrane thereby preventing the active efflux of doxorubicin and enhancing its cytotoxic activity. ABC, ATP-binding cassette.

Utilizing both of these distinct models, we observed that the semi-synthetic *Salmonella* nanoparticle mimic profoundly increases the efficacy of the chemotherapeutic drug, doxorubicin, as a way of reducing the required dose of this agent. It is notable that the doxorubicin dose administered in the humanized mouse model of primary breast cancer was fivefold lower than the dose delivered in the murine model colorectal carcinoma. In addition, the semi-synthetic *Salmonella* nanoparticle mimic targets to tumours, may not require access to the cytoplasm for efficacy, and induces functional suppression of P-gp by activation of a caspase-3-dependent degradation pathway (for this efflux pump; see model depicted in Fig. 8, and Hallstrom KN *et al.*^17^). Since the semi-synthetic *Salmonella* nanoparticle mimic attacks two different targets that are independently defined as major properties of cancer cells required to achieve sustain growth, such properties may offer an advantage over conventional therapeutic approaches. Repeated treatments of the semi-synthetic *Salmonella* nanoparticle mimic could also potentially trigger an untoward immune response, and current investigations are focused on determining the precise SipA domain responsible for the modulation of P-gp.

Our findings represent an important step forward in demonstrating the potential of this strategy as a ‘stand alone’ approach to increase cancer cell sensitivity to conventional chemotherapeutics. Indeed, a fundamental observation driving the initiative to develop a semi-synthetic *Salmonella* nanoparticle mimic is the observation that SipA appears to be broad spectrum since this effector protein is able to modulate P-gp expression in several cancers that are known to over-express P-gp (for example, colon, kidney, breast and lymphoma). Our observations showing that SipA downregulates the expression of P-gp in diffuse large B-cell lymphoma are especially significant, given that overexpression of this efflux transporter severely limits the use of numerous first-line anticancer drugs, such as doxorubicin currently used in the treatment of DLBCL. It is also tempting to speculate that SipA might offer an advantage with respect to previously developed small molecule entities that target MDR because this virulence determinant is a stable molecule that has co-evolved with the human host, and thus may coopt host machinery not attainable by other drug-based methods. In summary, engineering of this semi-synthetic *Salmonella* nanoparticle mimic introduces a new platform technology that can also be applied to various chemotherapeutic drugs to overcome MDR in tumours.

## Methods

### Chemicals

Anti-P-gp mouse mAb C219 and C494 were purchased from CalBiochem (La Jolla, CA). The CASP3 inhibitor (SC-3075) and CASP1 inhibitor (SC-3071) were purchased from Santa Cruz Biothechnology (Santa Cruz, CA). The Anti-HA affinity matrix and HA peptide were purchased from Roche Applied Science (Mannhein, Germany). Except where noted, all the chemicals for gold nanoparticles and ligand synthesis were purchased from Sigma-Aldrich.

### Cell culture

The human intestinal adenocarcinoma cell line HCT8 was obtained from ATTC and maintained in accordance with ref. 11. The human breast adenocarcinoma cell line MCF-7, the human bladder transitional cell carcinoma cell line UM-UC-3, and the CT26 murine colon carcinoma cell line, were purchased from ATCC and were all maintained in DMEM F-12 containing a 10% fetal bovine serum, 100 U ml^−1^ penicillin, and 10 μg ml^−1^ streptomycin at 37 °C in 90% relative humidity and 5% CO_2_. Diffuse large B-cell lymphoma cell line SU10 was obtained from ATTC and maintained in RPMI 1640 containing a 10% fetal bovine serum, 100 U ml^−1^ penicillin, and 10 μg ml^−1^ streptomycin at 37 °C in 90% relative humidity and 5% CO_2_.

### Bacterial strains and plasmids

*S.* Typhimurium ΔSipA, ΔSipB, ΔSipC and ΔSopB are derived from SL1344 (refs 14, 16). The AKJ63 strain has been previously described^44^. Briefly, the *sipA*-hemagglutinin (HA) gene fusion was constructed by using the pBH vector (Boehringer Mannheim). A HindIII–EcoRI DNA fragment containing the entire *sipA* ORF was prepared by PCR amplification. The *sipA* fragment was first cloned into pBluescript SK (Stratagene) to generate pAK62A. The *sipA* fragment then was subcloned into pBH to generate pAK68C. Plasmids were passaged through the r^−^ m^+^ *S.* Typhimurium strain LB5000 before being transformed into SL1344 to generate AJK63 (ref. 44).

### Isolation of S. Typhimurium-secreted proteins

Nonagitated microaerophilic cultures of wild-type *S.* Typhimurium SL1344 or its isogenic mutant derivatives were prepared by inoculating 30 ml of LB broth with 0.03 ml of a stationary-phase culture, followed by overnight incubation at 37 °C with ampicillin (50 μg ml^−1^) added to the cultures. The culture supernatants were collected and filtered through a 0.22-μm filtre (MIllipore). The proteins from the supernatants were precipitated with 15% (vol/vol) trichloroacetic acid, solubilized in 1 ml of 100% acetone, and then centrifuged at 15,000 r.p.m. for 5 min. The protein pellets are dried and then resuspended in PBS buffer.

### The purification of SipA-HA fusions protein

The purification of SipA was performed in accordance with the work of Lee *et al.*^44^ with minor modifications. Brifely, nonagitated microaerophilic cultures of the AJK63 strain were prepared by inoculating 30 ml of LB broth with 0.03 ml of a stationary-phase culture, followed by overnight incubation at 37 °C with ampicillin (50 μg ml^−1^) added to bacterial culture media. The culture supernatants were collected and filtered through a 0.22-μm filtre (Millipore). The SipA-HA recombinant fusion protein was purified by passing the supernatant through a 0.5 ml HA-affinity matrix column (Roche, Mannhein, Germany). To elute the bound protein, 1 mg of the HA peptide was dissolved in 1 ml of the column buffer and was subsequently passed through the column.

### Cell lysates and P-gp western blot analysis

Cell lysates were collected from either *S.* Typhimurium-infected or purified-SipA exposed to HCT8 cells, as previously described^11^. Proteins were normalized to 30 μg, separated by SDS/PAGE (4−12% gradient; Biorad, Hercules, CA), and transferred to nitrocellulose (Bio-Rad; 0.45 μ membrane). Immunoblots were performed using the murine monoclonal P-gp C219 antibody (Calbiochem Cat. No. 5173310) diluted at 1:100. Glycaraldehyde-3-phosphate dehydrogenase (1:1,000; Millipore, Temecula, CA) was used as a loading control. A goat anti-mouse IgG labelled with horseradish peroxidase (Santa Cruz, CA) diluted at 1:10,000 was used to detect the bands, which were visualized by enhanced chemiluminescence using a super signal West pico kit (Thermo, Rockford, IL). Uncropped scans of the western blots images related to Figs 1a,c; 2a and 3b,d, [Fig f3] are found in the Supplementary Information (Supplementary Fig. 17).

### *In vitro* P-gp cleavage

To assess *in vitro* P-gp cleavage, we followed the method of Mantovani, *et al.*^15^. Briefly, HCT8 cells were infected, as previously described^11^. Following infection, the cells were washed twice in PBS, containing the complete protease inhibitor cocktail (Roche Applied Science, Indianapolis, IN) and the phosphatase inhibitor cocktail (1 mM Na_3_VO_4_, 2.5 mM Na pyrophosphate, 1 mM 2-glycerolphosphate, 25 mM NaF). Cells were then lysed in boiling electrophoresis sample buffer containing the protease and phosphatase inhibitor cocktails. Lysates were sonicated to shear DNA and reduce viscosity, and boiled for 5 min to solubilize proteins. Samples were separated by SDS/PAGE (4–12% gradient; Biorad, Hercules, CA), and transferred to nitrocellulose (Bio-Rad; 0.45-μm membrane). Immuno-blots were performed using the murine monoclonal P-gp C494 antibody (Calbiochem Cat. No. 517312) diluted at 1:1,000. A goat anti-mouse IgG labelled with horseradish peroxidase (Santa Cruz, CA) diluted at 1:10,000 was used to detect the bands, which were visualized by enhanced chemiluminescence using a super signal West pico kit (Thermo, Rockford, IL).

### Cell proliferation assays

Cytotoxicity assays were performed as described previously^45^ with minor modifications. HCT8 cells (2,500 per well) were plated in 96-well Cellbind plates (Corning, Tewksbury, MA) in 100 μl of growth media. After overnight attachment, cells were incubated for 72 h with doxorubicin (1 μg ml^−1^) and purified SipA (100 μg ml^−1^), alone and in combination. Verapamil (20 μM) was used as a positive control, alone and in combination with doxorubicin. After treatment, the number of viable cells was determined by a colorimetric cell proliferation assay (CellTiter96 Aqueous One solution; Promega, Madison, WI) according to the manufacturer’s instructions. All studies were conducted in triplicate and performed at least three times independently.

### Drug response curves

Cytotoxicity assays were performed as described previously^45^,^46^,^47^ with minor modifications. HCT8 cells (2,500 per well) were plated in 96-well Cellbind plates (Corning, Tewksbury, MA) in 100 μl of growth media. After overnight attachment, the culture medium was replaced with media containing different concentrations of doxorubicin, vinblastine, or 5-FU—ranging from 0.001 to 100 μM—with or without purified-SipA (80 μg ml^−1^) and incubated for 72 h. After treatment, cell viability was determined by a colorimetric cell proliferation assay (CellTiter96 Aqueous One solution; Promega, Madison, WI) according to the manufacturer’s instructions. Dose–response curves were plotted as percentages of the control cell absorbance (wells without doxorubicin). IC_50_ values were calculated from dose–response curves obtained from at least three independent experiments using the GraphPad Prism (GraphPad Software).

### Intracellular doxorubicin accumulation

HCT8 cells were seeded on 12-mm glass slides at ∼3 × 10^4^ and kept in standard 24-well plates (Corning, Corning, NY). Approximately 18 h after seeding, the culture medium was replaced by medium containing doxorubicin (0.01 μg ml^−1^), purified-SipA (80 μg ml^−1^) combined with doxorubicin (0.01 μg ml^−1^), purified-SiFA (80 μg ml^−1^) combined with doxorubicin (0.01 μg ml^−1^), verapamil (20 μM) combined with doxorubicin (0.01 μg ml^−1^) or left in culture medium. After 24 h, the glass slides were washed in 1% PBS, fixed with 1% paraformaldehyde (PFA) in PBS for 15 min, quenched with NH_4_Cl in PBS for 15 min, and then washed again in 1% PBS. Imaging was performed with a Nikon A1 confocal microscope using a × 60 objective with a pinhole size of 0.9 a.u. resulting in a 0.36-μm depth of field. Due to the fluorescence nature of doxorubicin, excitation was carried out with the 488-nm laser line and emission was detected between 565 and 630 nm using the TRITC filtre cube. DIC images were also simultaneously acquired. All imaging settings were kept consistent across all samples in an experiment.

### Syntheses of the ligand (dithiolated TEG carboxylic acid)

Detailed synthesis scheme was illustrated in Supplementary fig. 7. Here compound 1 (Undec-l-en-11-yl-tetra (ethy1ene glycol)) and 2 (ethyl 3,6,9,12,15-pentaoxahexacos-25-en-1-oate) were synthesized according to the literatures, respectively^48^,^49^. To make the compound 3, at 0 °C, bromine (0.28 mmol) was added to a solution of ethyl 3,6,9,12,15-pentaoxahexacos-25-en-1-oate 2 (compound 2; 0.10 g, 0.23 mmol) in dry DCM (10 ml). The reaction mixture was stirred at 0 °C for 2 h in dark. Thereafter, the product was washed with saturated Na_2_SO_3_ solution and extracted with DCM. The organic layer was combined and subject for rotary evaporation. Further purification by silica chromatography give 0.138 g colourless oil, yield 95%. ^1^H NMR (400 MHz, CDCl_3_) *δ*=1.22–1.38 (m, 13H), 1.49–1.61 (m, 4H), 1.71–1.80 (m, 1H), 2.12–2.28 (m, 1H), 3.42–3.47 (t, 2H), 3.58–3.77 (m, 18H), 3.81–4.87 (m, 1H), 4.17 (s, 2H), 4.19–4.22 (m, 2H). ^13^C NMR (100 MHz, CDCl_3_) *δ*=170.45, 70.62, 70.60, 70.58, 70.54, 70.03, 68.70, 60.79, 53.15, 36.37, 35.98, 29.43, 29.41, 29.30, 28.77, 26.72, 26.05, 14.21. MS (ESI-MS) calculated for C_23_H_44_Br_2_O_7_ 592.4, found 593.0 [M+H]^+^.

A solution of dibromine (compound 3; 100 mg, 0.17 mmol) and K_2_CO_3_ (117 mg, 0.85 mmol) in acetone (10 ml) was added thioacetic acid (129 mg, 1.7 mmol). The reaction mixture was stirred at room temperature overnight. The solvent was removed by using a slight vacuum and then the product was purified by chromatography on silica gel (eluent: ethyl acetate/hexane) to give 22.6 mg ethyl 25-(acetylthio)-28-oxo-3,6,9,12,15-pentaoxa-27-thianonacosan -1-oate (compound 4) as light yellow oil, yield 21%. ^1^H NMR (400 MHz, CDCl3) *δ*=1.09–1.35 (m, 13H), 1.48–1.68 (m, 4H), 1.93–2.01 (m, 2H), 2.32 (s, 6H), 3.39–3.50 (m, 4H), 3.55–3.78 (m, 17H), 4.15 (s, 2H), 4.18–4.24 (m, 2H). ^13^C NMR (100 MHz, CDCl3) *δ*=195.56, 170.46, 71.51, 70.86, 70.62, 70.60, 70.58, 70.53, 70.02, 68.70, 60.80, 32.22, 30.51, 29.60, 29.40, 29.38, 29.02, 26.05, 14.21. MS (ESI-MS) calculated for C_27_H_50_O_9_S_2_ 582.3, found 600.4 [M+H_3_O]^+^.

The solution of 10 mg diactyl-OEt (compound 4) in 2 ml ethanol was then added with concentrated hydrochloric acid (0.5 ml, 6 M) and stirred overnight. The solvent was removed by using a slight vacuum and dried to give 8 mg (dithiolated TEG carboxylic acid) (compound 5), yield 96%. ^1^H NMR (400 MHz, CDCl3) *δ*=1.18–1.38 (m, 15H), 1.49–1.61 (m, 2H), 3.36–3.42 (t, 2H), 3.55–3.77 (m, 18H), 4.17 (m, 2H), 4.61–4.78 (br, 2H). ^13^C NMR (100 MHz, CDCl_3_) *δ*=170.56, 71.53, 70.81, 70.55, 70.51, 70.47, 69.98, 68.67, 60.87, 58.39, 29.59, 29.46, 26.07, 18.37. MS (ESI-MS) calculated for C_21_H_42_O_7_S_2_ 471.2, found 473.4 [M+H]^+^.

For NMR and MS analysis of the compounds (3–5), see Supplementary Figs 8–16.

### Syntheses of the *Salmonella* nanoparticle mimic

Fifteen-nanometre sized AuNPs were first synthesized using citrate as a reducing agent and stabilizer. HAuCl_4_ (10 mg) was dissolved in 90 ml of water, and the solution was heated to the boiling point. Sodium citrate solution (500 μl of 250 mM) was added to the boiling solution and stirred for 30 min until the colour turned to wine red. The resulting AuNP was then centrifuged and washed three times. The concentration of afforded nanoparticle was estimated according to the method described in the literature by using the diameter of the gold nanoparticles and absorption at 450 nm (ref. 50). Five milligram of the dithiolated TEG carboxylic acid (compound 5) was subsequently mixed with 10 pmoles of AuNPs in 5 ml of water, leading to an overnight ligand change reaction. The afforded Au-COOH nanoparticles were dialyzed in deionized (DI) water using a Slide-A-Lyzer MINI dialysis unit (COMW=10,000) for 2 days. We then conjugated and characterized the SipA AuNPs.

Typically, 1-Ethyl-3-(3-dimethylaminopropyl) carbodiimide 10 mg and N-hydroxysuccinimide 10 mg were added to the solution of Au-COOH (10 pmoles). The resultant mix was stirred at room temperature. After 1 h, the solution was centrifuged, washed three times with DI water, and concentrated to 2 ml. Following, 200 μl (540.7 μg ml^−1^) of SipA-HA mix was added to this solution and stirred at 4 °C for12 h. After this period, the solution was centrifuged, washed three times with DI water, and the SipA-AuNP stock solution was concentrated to 0.5 ml of DI water and was then dialysed with Slide-A-Lyzer dialysis unit (COMW=100,000) in PBS buffer overnight. The stability of the *Salmonella* nanoparticle mimic was evaluated by monitoring aggregation. Suspensions of the SipA-AuNP in DI water were placed in clear microcentrifuge tubes and stored at 5 °C, and no observable aggregation was detected at two different time points following conjugation (7 and 15 days); a time frame consistent with the duration of the *in vivo* experiments.

### The subcutaneous tumour model

Eight- to 10-week-old female BALB/c mice (*n*=6 per group) were purchased from Jackson Laboratory (Bar Harbor, ME) and allowed to acclimatize for 4 days. CT26 cells were collected by trypsin treatment, washed twice in PBS buffer, and resuspended in PBS. CT26 cells (5 × 10^5^) were inoculated in 100 μl subcutaneously into the right flank^34^. Mice were randomly assigned to the control group or the treatment groups. After several days, the mice harboured tumours with volumes of ∼0.5 mm^3^, and were i.p. injected with 2.5 pmoles per day of SipA-AuNP (200 μl, containing 1.1 μg SipA) for 2 days. The following day, the mice received a one-time drug treatment of doxorubicin (10 mg kg^−1^) delivered by i.p. injection, followed by 2.5 pmoles per day of SipA-AuNP (i.p.) every 48 h for 15 days. Two weeks post drug delivery the mice were killed and the tumours were extracted for analysis, in regard to tumour size and expression of P-gp. tumour size was measured using calipers and volumes were estimated using the formula 0.5 × length × (width)^2^. The care of these animals was in accordance with University of Massachusetts Medical School institutional guidelines under protocol number: 2046-12. Statistical analysis was performed using Prism software (GraphPad).

### Mouse infections

Mouse infections were performed as previously described^14^. Briefly, 8–10-week-old female BALB/c mice (*n*=6) were purchased from Jackson Laboratory (Bar Harbor, ME) and allowed to acclimatize for 4 days. Before infection, mice were given 3.75 mg of streptomycin intragastrically. The following day, water and food were withdrawn for 4 h before oral inoculation with 5 × 10^7^ CFU of *S.* Typhimurium SL1344 or its isogenic mutant derivatives.

### Apoptosis assay (Annexin V)

HCT-8 cells grown on 6-well plates (Costar) were either treated with the apoptosis inducer staurosporine (0.5 mg ml^−1^) or treated with either 160 μg or 320 μg ml^−1^ of SipA-HA for 3 h at 37 °C. The plates were subsequently treated with 0.05% trypsin for 10 min at 37 °C. Cells were then removed from plates, washed with cold PBS and 2 × 10^6^ cells from each sample were prepared for FACS analysis using the Annexin V-FITC apoptosis detection kit (Santa Cruz Biotechnology, Dallas, TX).

### Biodistribution

Eight- to 10-week-old female BALB/c mice (*n=*6) were purchased from Jackson Laboratory (Bar Harbor, ME) and allowed to acclimatize for 4 days. CT26 colon carcinoma cells were implanted as described above for the subcutaneous tumour model. BALB/c mice bearing subcutaneous CT26 tumours (mean tumour volumes of ∼0.5 mm^3^) received i.p. treatments of the SipA-AuNP every 48 h for 15 days. At necropsy, brain, spleen, heart, kidney, lung, liver and tumour tissues were collected and snap-frozen into liquid nitrogen. Tissue samples were subjected to elemental analysis (Elemental Analysis Inc., Lexington KY.

### SEM and X-ray microanalysis of histological sections

Hemotoxylin and eosin stained sections of tumours, provided by the University of Massachusetts’ Histopathology Core facility were soaked in xylene to remove the coverslips. Once the coverslips were removed, the slides were transferred through three changes of 100% ethanol to remove the xylene, and then transferred to *N*-amyl acetate for 30 min and allowed to air dry. Now, the dry tumour sections were mounted onto scanning electron microscopic (SEM) stubs with carbon conductive tape and grounded to the stubs with strips of pure copper tape. The mounted slides were then carbon coated (3 nm) in a Denton High Vacuum evaporator. The sections of tumour were imaged on a FEI Quanta 200 FESEM equipped with an Oxford-Link EDS (energy dispersive spectrometer) for X-ray analysis and X-ray mapping. All X-ray maps were collected using 10 kV accelerating voltage and imaged at × 100 magnification with the X-ray emission window set at 2.1205, keV (the energy window for the Au, Ma1 emission line). X-ray maps were collected and averaged over 10 sequential scans using Oxford-Link INCA software and displayed as Cameo composite images (X-ray map overlay on the SEM image) with the intensity of the X-ray emission displayed as an intensity map (blue to red-white).

### Humanized model of primary breast carcinoma

Eight- to 10*-*week-old female immunodeficient NOD-*Rag1*^*Tm1Mom*^ *IL2rgTm1Wjl/Sz* (NRG) mice were supplied by Jackson Laboratory (Bar Harbor, ME) and allowed to acclimatize for 4 days (*n=*6 per group). Primary breast cancer tumours were provided by The UMass Cancer Avatar Institute (IRB ID: H00004721). Tumour fragments of ∼1 mm^3^ were implanted into the mammary fat pad using a 10-gauge sterile trocar. Mice were randomly assigned to the control group or the treatment groups. Within 1–2 weeks, the mice harboured tumours with volumes of ∼3 mm^3^, and were i.p. injected with 2.5 pmoles per day of SipA-AuNP (200 μl, containing 1.1 μg SipA) for 2 days. The following day, the mice received a one-time drug treatment of doxorubicin (2 mg kg^−1^) delivered by i.p. injection, followed by 2.5 pmoles per day of SipA-AuNP (i.p.) every 48 h for 33 days. Two weeks post drug delivery the mice were killed and the tumours were extracted for analysis, in regards to tumour size and expression of P-gp. Tumour size was measured using calipers and volumes were estimated using the formula 0.5 × length × (width)^2^. The care of these animals was in accordance with University of Massachusetts Medical School institutional guidelines under protocol number: 2046-12. Statistical analysis was performed using Prism software (GraphPad).

### Statistical analysis

Western blots are presented as one representative of at least three experiments showing reproducible results. Densitometry was analysed by ImageJ and presented as relative to the untreated cells (mean±s.d.). *P* values were calculated using the Student’s *t*-test, and values of <0.05 were considered statistically significant. All other quantitative results were analysed by one-way analysis of variance and presented as means±s.d. For all comparisons, a *P* value of<0.05 was considered significant.

## Additional information

**How to cite this article:** Mercado-Lubo, R. *et al.* A *Salmonella* nanoparticle mimic overcomes multidrug resistance in tumours. *Nat. Commun.* 7:12225 doi: 10.1038/ncomms12225 (2016).

## Supplementary Material

Supplementary Information Supplementary Figures 1-17 and Supplementary Reference
