# Supplementary material for: A Salmonella nanoparticle mimic overcomes multidrug resistance in tumours
Source: Nat Commun. 2016 Jul 25;7:12225. doi: 10.1038/ncomms12225 (PMC5512628; doi:10.1038/ncomms12225)
Supplement: Supplementary Information — Supplementary Figures 1-17 and Supplementary Reference [file ncomms12225-s1.pdf]

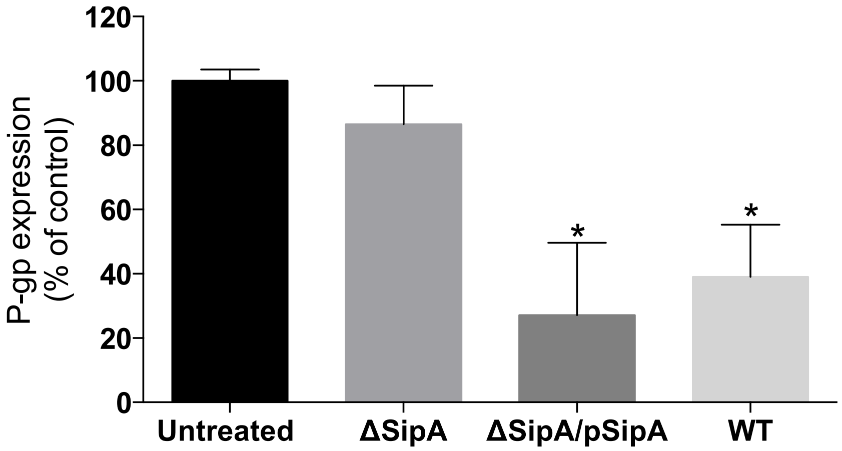

Supplementary Figure 1. SipA modulates the expression of P-gp in healthy murine intestinal epithelium in vivo. *Salmonella* Typhimurium AJK63 (ΔSipA/pSipA) was able to modulate the expression of P-gp in healthy murine intestinal epithelium *in vivo*.

Balb/c mice were infected with 10^7 CFU of either SL1344 ΔSipA, SL1344 ΔSipA complemented with SipA (ΔSipA/pSipA) or SL1344 WT for 48 hours, after which the proximal colon was dissected, homogenized and lysed. Whole cell lysates were normalized for protein levels and probed for P-gp. Levels of P-gp were quantified by densitometry and presented on the bar graph. Densitometry was performed using ImageJ and results are presented as relative to the untreated cells (n=6). Data represent means ± SD. * P<0.0001 (Student's t-test).

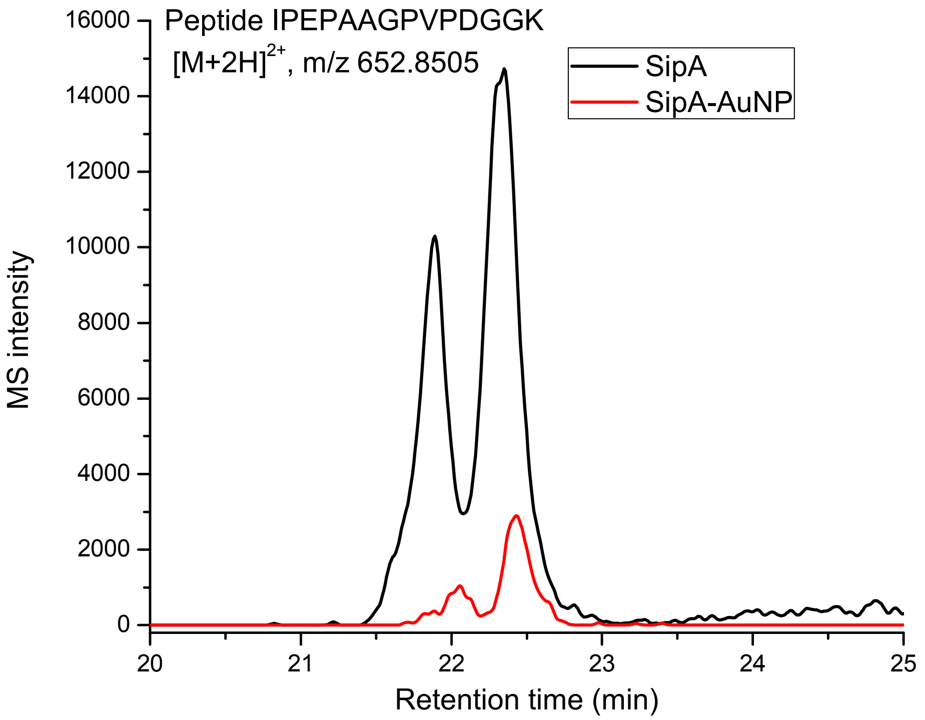

Supplementary Figure 2. Determination of the ratio of AuNP to surface conjugated SipA proteins. The extracted ion chromatograph (EIC) peaks for peptide IPEPAAGPVPDGGK from the SipA and SipA-AuNP samples. The number of moles of SipA that was conjugated on AuNP ratio was estimated to be 1:6.

To determine the ratio of AuNP to corona SipA proteins, we exposed the SipA-AuNP (7.2 pmoles, estimated by UV-Vis absorption and the diameter of nanoparticle[1]) sample to sodium cyanide, which decomposes the gold particle core. This afforded solution was then dialyzed using a Slide-A-Lyzer MINI dialysis unit (MW=10,000), and thereafter concentrated to 45μl. This sample and the same volume Sip A only (45μl, 540.7 μg/ml) sample are trypsin digested and measured with an Agilent Q-TOF 6538 mass spectrometer coupled with an Agilent HPLC 1200. Peptide IPEPAAGPVPDGGK ($[M+2H]^{2+}$, m/z 652.8505) from the SipA protein is identified through MS/MS spectral match, and chosen for as surrogate for protein quantification. The extracted ion chromatograph (EIC) peaks for this peptide from the

aforementioned two samples are integrated and compared. (Figure S1). The ratio protein only /protein from SipA-AuNP is 7.5 based on the integrated areas.

The ratio of AuNP and conjugated SipA from SipA-AuNP can be determined by the following equations:

(1) The total mass weight of SipA that was conjugated on AuNP is :

$(45\ \mu l \times 540.7\ \mu g/ml)/7.5 = 3.24\ \mu g$

(2) The number of moles of SipA that was conjugated on AuNP is :

$3.24\ \mu g/\ 74{,}000 g/mol$ (MW of SipA)= 43.8 pmoles

Thus the ratio of AuNP and conjugated SipA is estimated to be 1:6 (7.2pmols vs. 43.8 pmols)

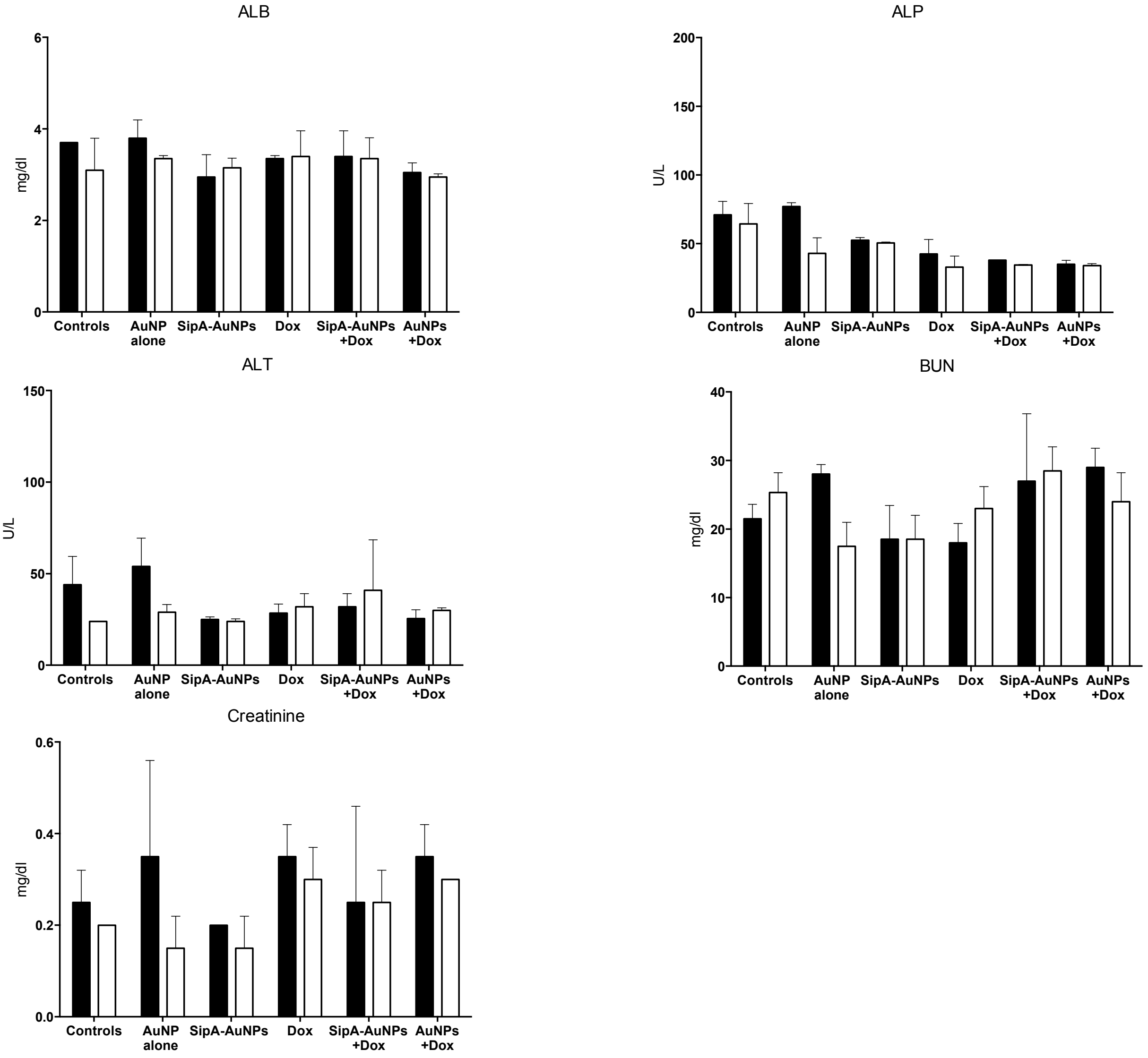

Supplementary Figure 3. Liver enzymes and blood chemistries remained within normal limits throughout the duration of the experiment: (Balb/c CT26 model). After a full course treatment (every 48 hours for 15 days) with 2.5 pmoles of SipA-AuNP liver enzymes and kidney function were found within normal limits. Day 1 (black bars) or day 15 (white bars).

To determine the blood chemistry profiles blood samples (100 to 125 µl) were obtained by incising the right facial vein with a sterile 5 mm lancet (MediPoint, Mineola, NY) from female 8 to 10 week old Balb/c mice. Results for concentrations of albumin, alkaline phosphatase (ALP), alanine aminotransferase (ALT), creatinine, and blood urea nitrogen

(BUN) were obtained from the VetScan Classic (Abaxis, Union City, CA) following manufacturer instructions. Values indicate mean ± SD (n = 3).

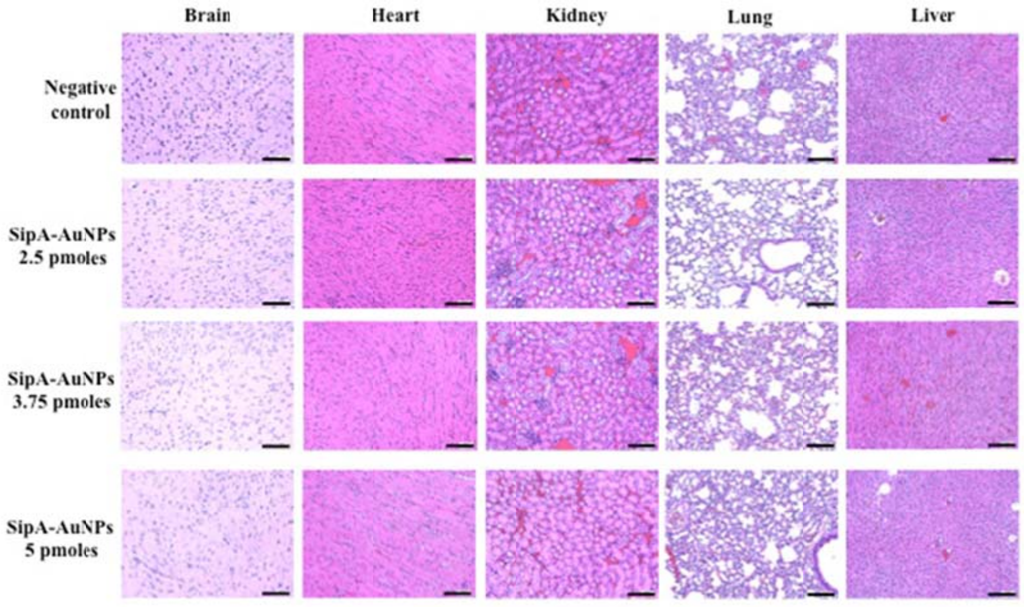

Supplementary Figure 4. No significant histological changes were detected in major organs following the treatment with an escalating dose of the SipA-AuNP. After a full course treatment (every 48 hours for 15 days) with escalating doses of SipA-AuNp, 2.5 pmoles (normal dose), 3.75 pmoles and 5 pmoles, histopathological changes were not found in major organs. Scale bars, 100 µm.

To find whether IP treatment with SipA-AuNP would induce histopathological changes in major organs, 8 to 10 week old female Balb/C mice were IP injected with one, one and a half or two times the normal dose of SipA-AuNP every 48 hours for 15 days. After 2 weeks mice were sacrificed and major organs (brain, heart, lung, liver and kidney) were extracted for histopathological examination. Tissues were collected, formalin-fixed, and embedded in paraffin. Four sections (5 µm) were mounted on glass slides and stained with hematoxylin and eosin (H&E). The sections were analyzed by a trained pathologist without prior knowledge of the type of treatment.

A

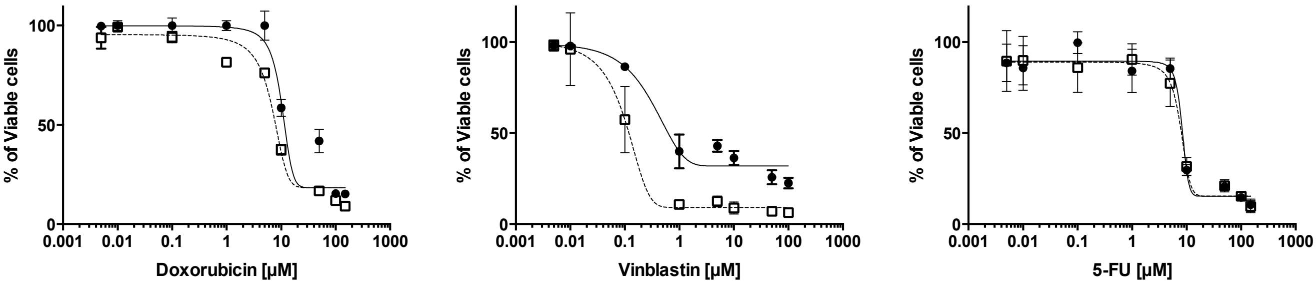

B

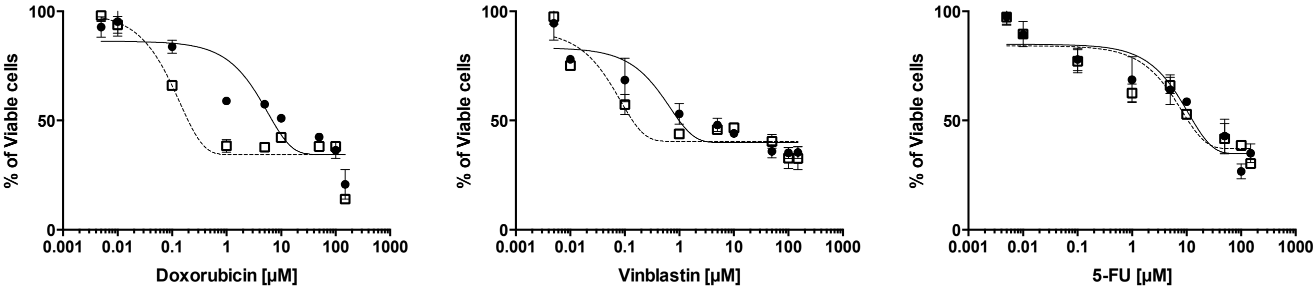

Supplementary Figure 5. SipA enhances the cytotoxic effect of P-gp substrates on UC-UM-3 and MCF7 cells.

Dose response curves of doxorubicin and vinblastine (P-gp substrates), as well as 5-FU (not a P-gp substrate) in the absence (●) or in the presence of purified-SipA (❐). UC-UM-3 and MCF7 cells were grown for 72 hours with specific concentrations of the cytotoxic drugs with or without purified-SipA (80 µg/ml). Cell viability was measure by CellTiter96 Aqueous One solution cell proliferation assay (*see methods*). (A) In UC-UM-3 IC_{50} shifted from 28.39 ± 3.83 µM to 8.85 ± 0.39 µM for doxorubicin and from 0.71 ± 0.063 µM to 0.1 ± 0.086 µM for vinblastine. No change in IC_{50} was observed for 5-fluorouracil. (B) In MCF7 cells, IC_{50} shifted from 8.86 ± 0.19 µM to 0.29 ± 0.26 µM for doxorubicin and from 1.05 ± 0.49 µM to 0.138 ± 0.019 µM for vinblastine. No change in IC_{50} was observed for 5-fluorouracil. Dose

response curves were derived from three independent experiments; error bars indicate ± SD (n = 3). Absent error bars indicate that error fell within symbol.

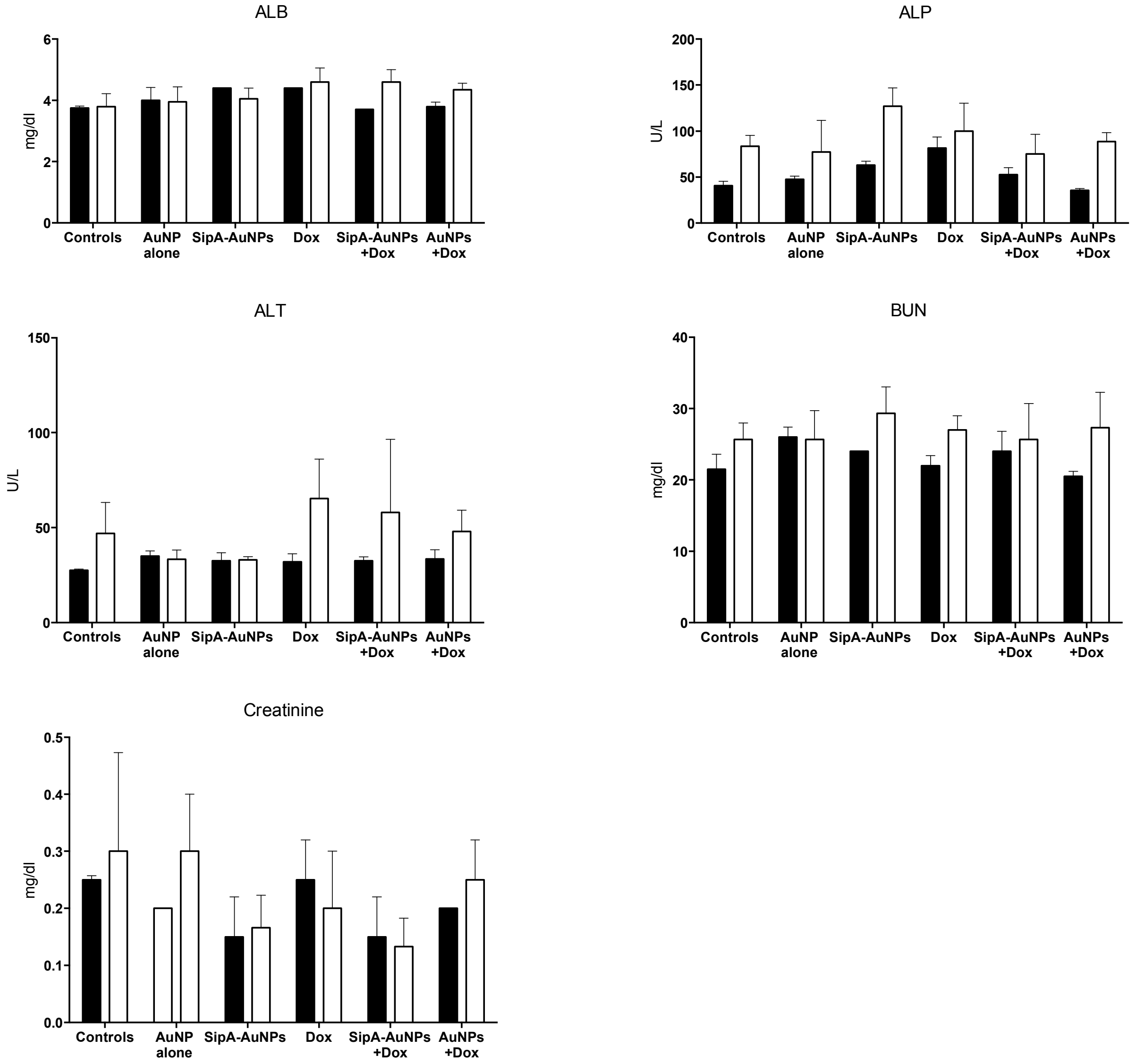

Supplementary Figure 6. Liver enzymes and blood chemistries remained within normal limits throughout the duration of the experiment: (NRG primary breast cancer model) After a full course treatment (every 48 hours for 33 days) with 2.5 pmoles of SipA-AuNP liver enzymes and kidney function were found within normal limits. Day 1 (black bars) or day 33 (white bars).

To determine the blood chemistry profiles blood samples (100 to 125 µl) were obtained by incising the right facial vein with a sterile 5 mm lancet (MediPoint, Mineola, NY) from female 8 to 10 week old Balb/c mice. Results for concentrations of albumin, alkaline phosphatase (ALP), alanine aminotransferase (ALT), creatinine, and blood urea nitrogen

(BUN) were obtained from the VetScan Classic (Abaxis, Union City, CA) following manufacturer instructions. Values indicate mean ± SD (n = 3).

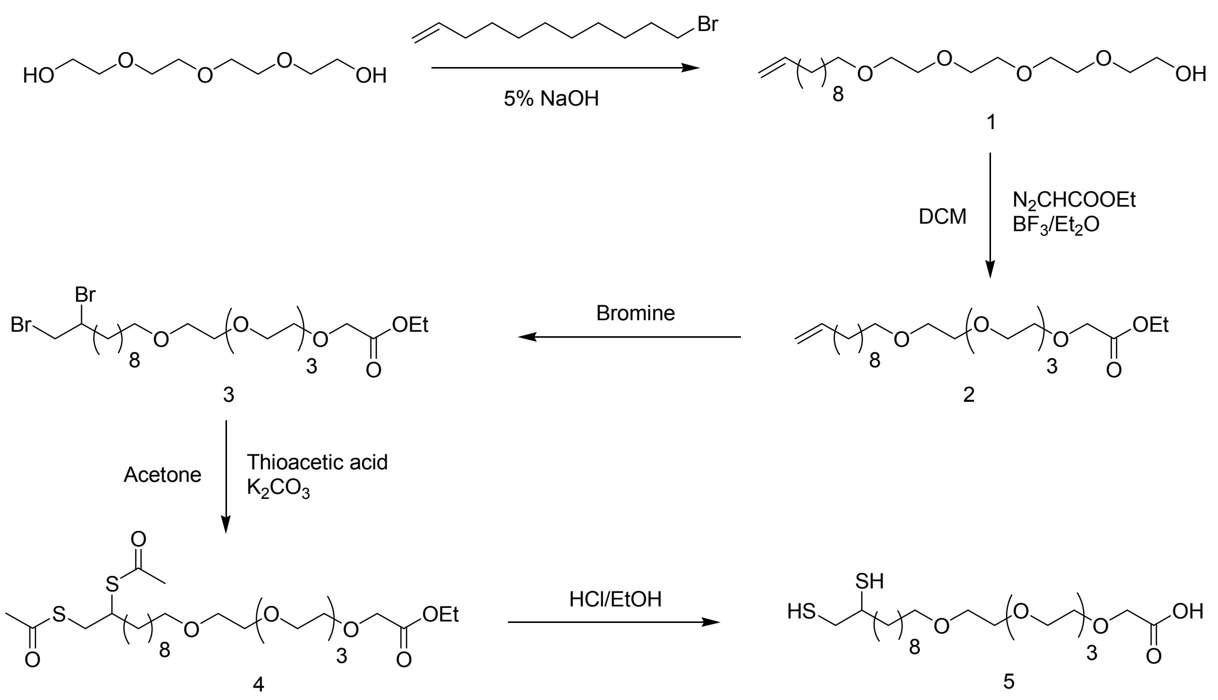

Supplementary Figure 7. Scheme of synthesis of the dithiolated tetra (ethy1ene glycol) carboxylic acid.

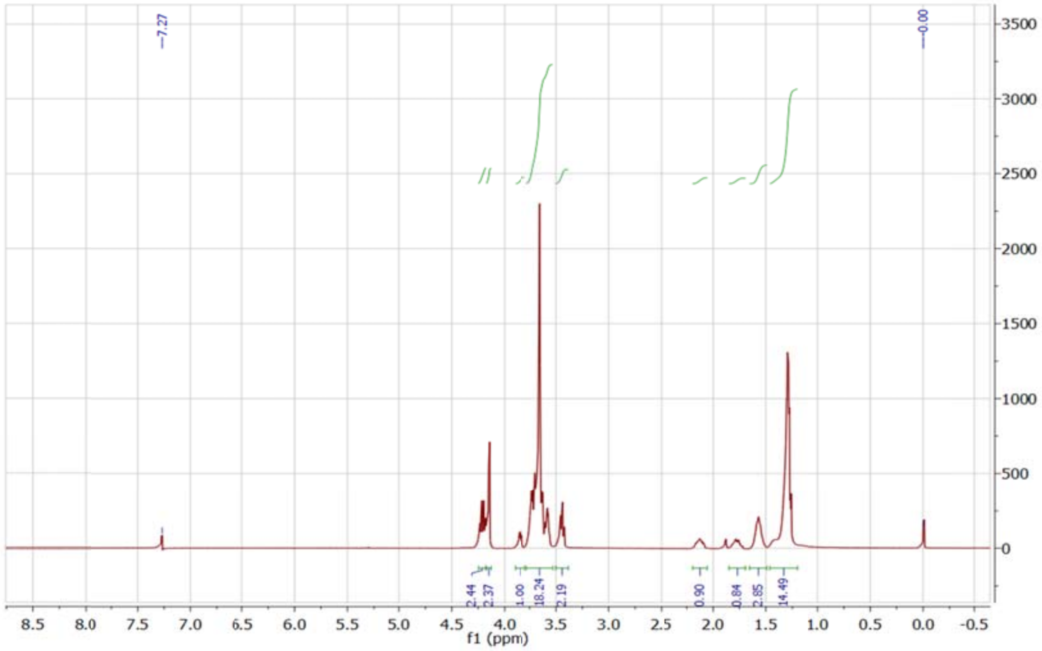

Supplementary Figure 8. ^{1}H NMR spectra of compound 3.

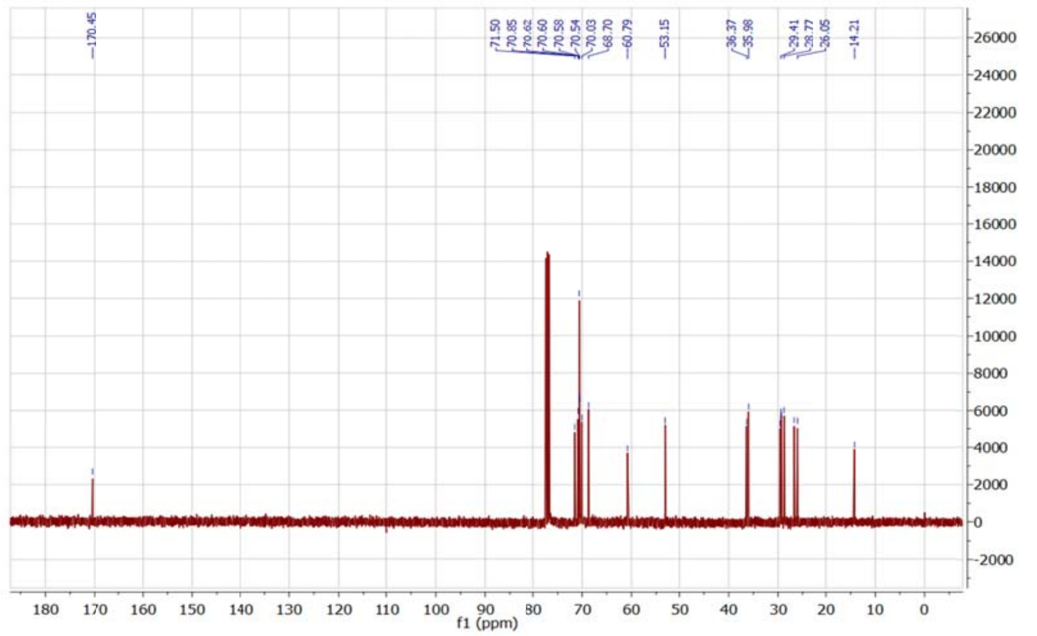

Supplementary Figure 9. ^{13}C NMR spectra of compound 3.

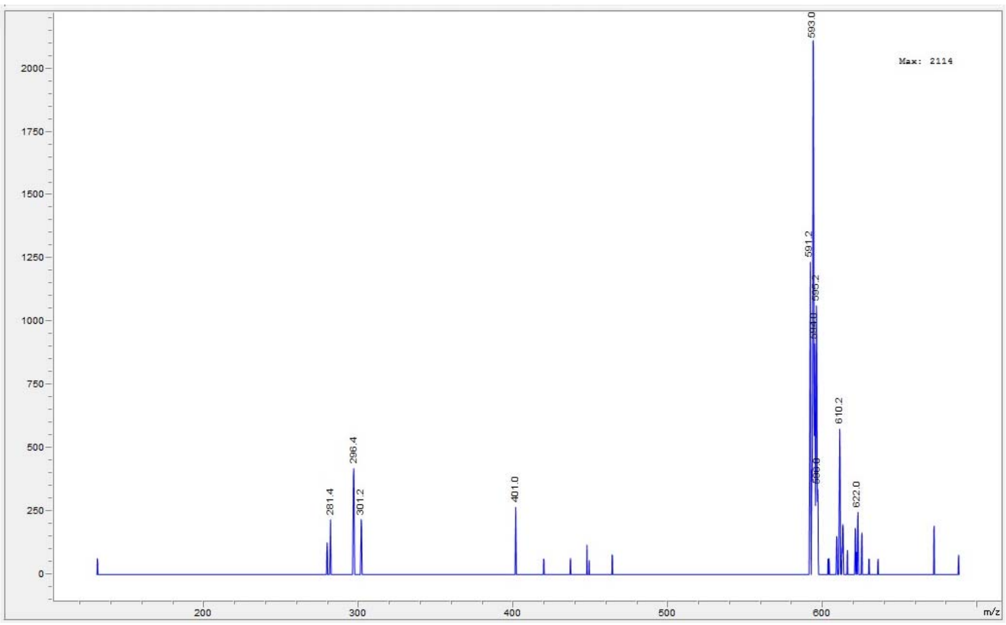

Supplementary Figure 10. MS spectra of compound 3.

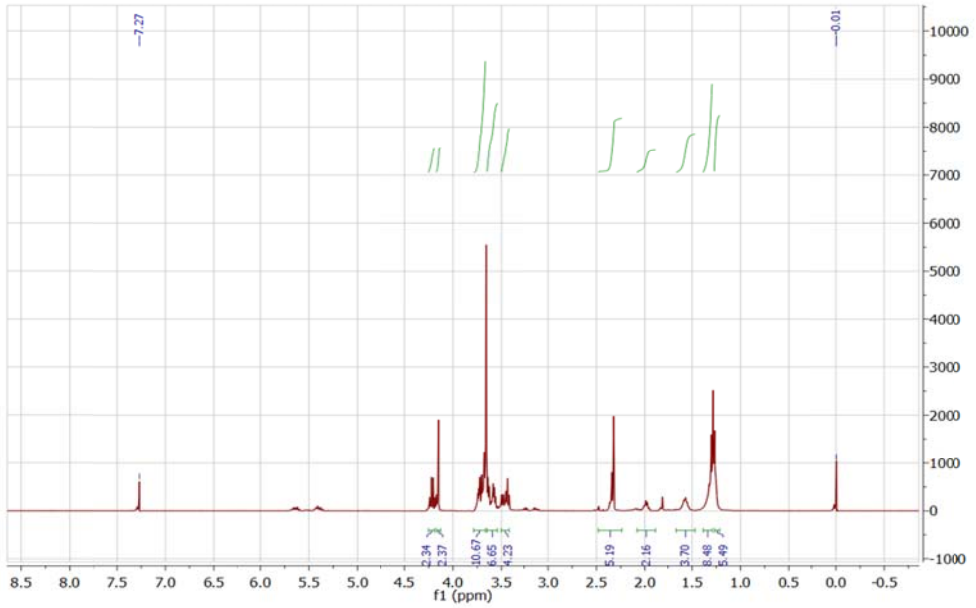

Supplementary Figure 11. ^{1}H NMR spectra of compound 4.

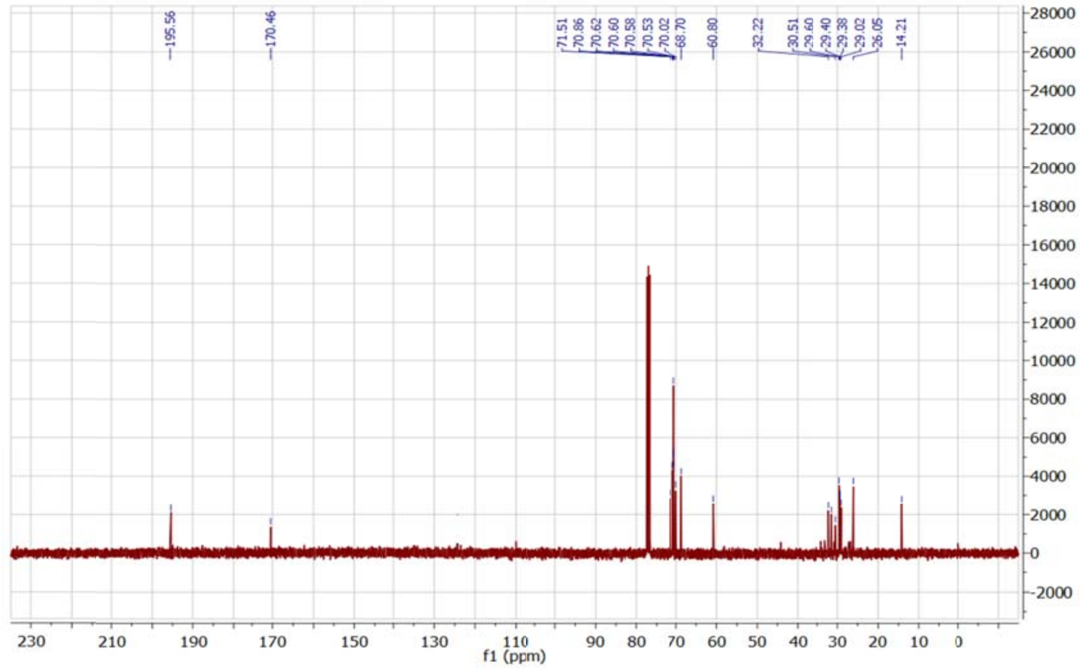

Supplementary Figure 12. ^{13}C NMR spectra of compound 4.

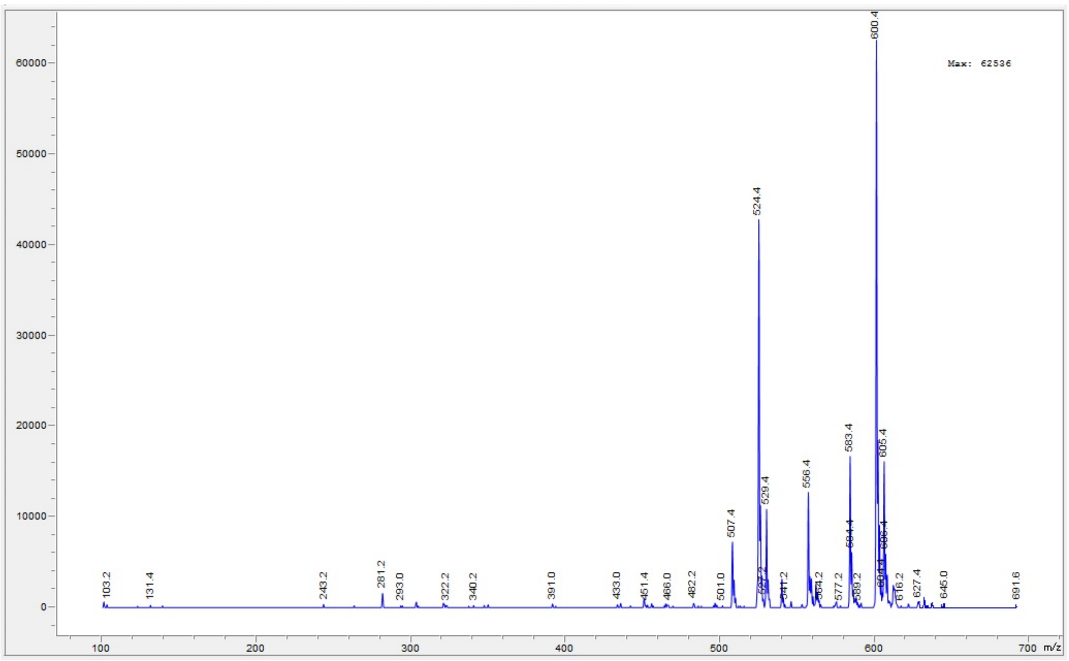

Supplementary Figure 13. MS spectra of compound 4.

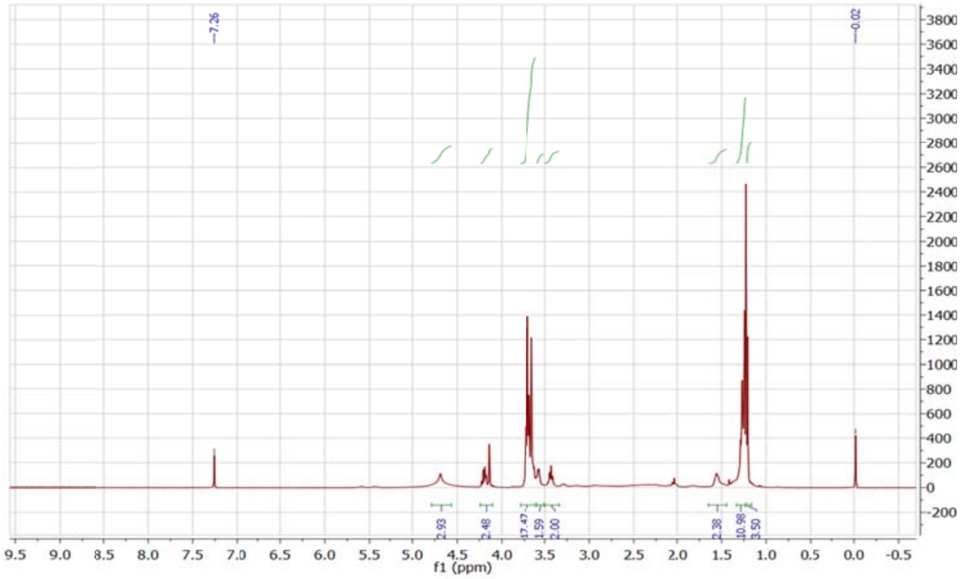

Supplementary Figure 14. ^{1}H NMR spectra of compound 5.

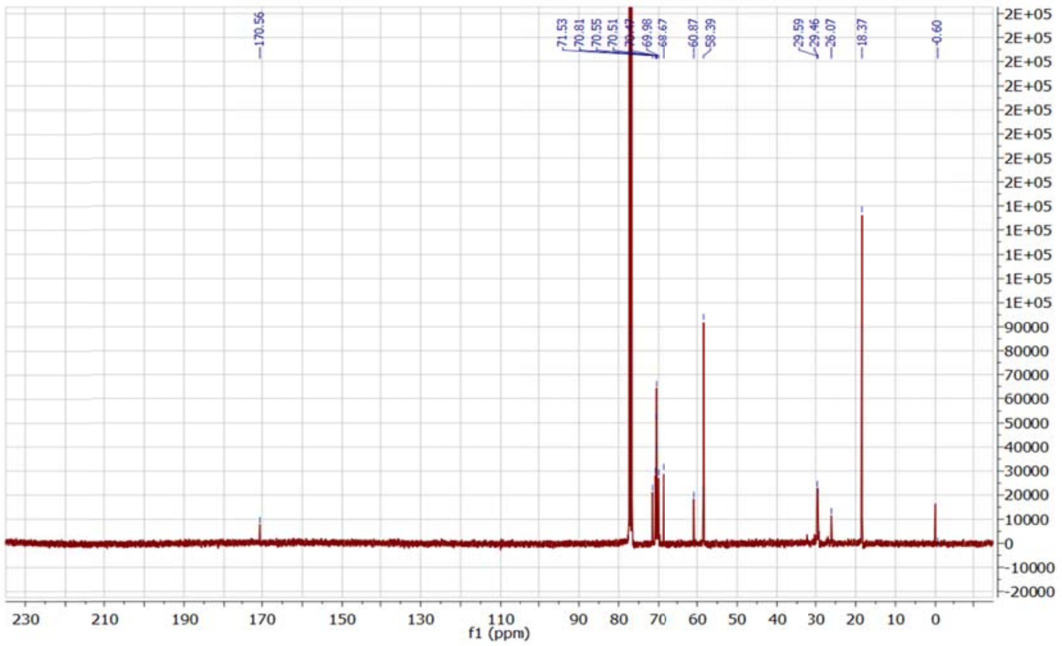

Supplementary Figure 15. ^{13}C NMR spectra of compound 5.

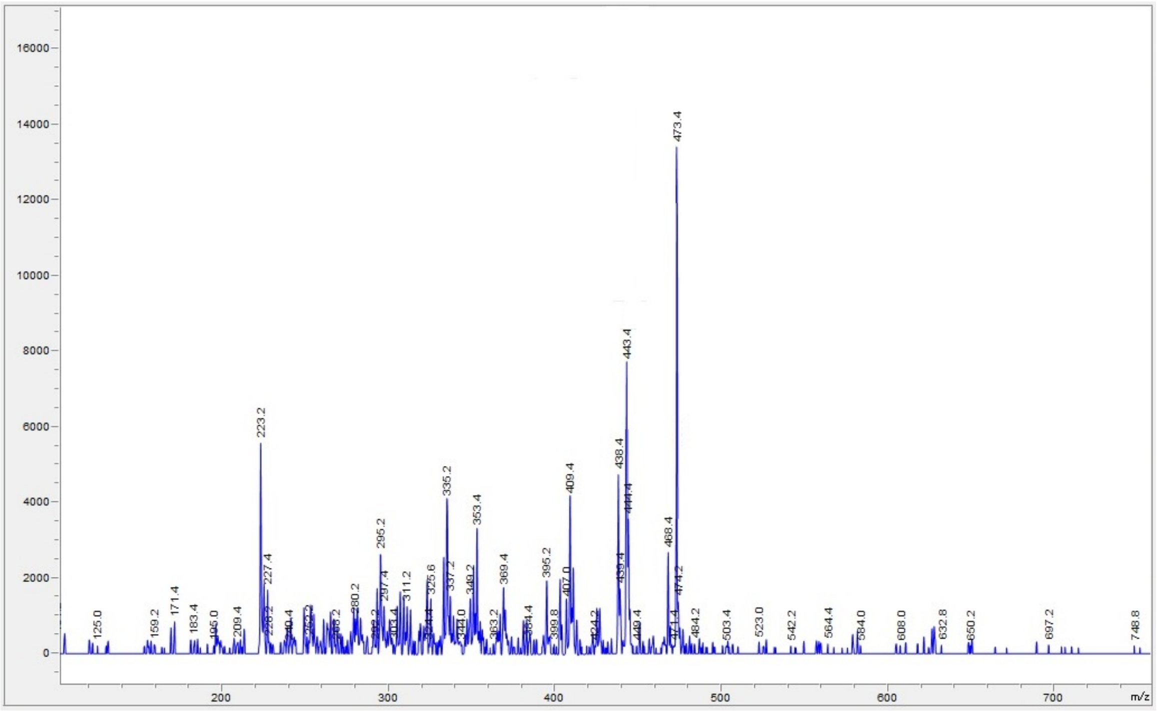

Supplementary Figure 16. MS spectra of compound 5.

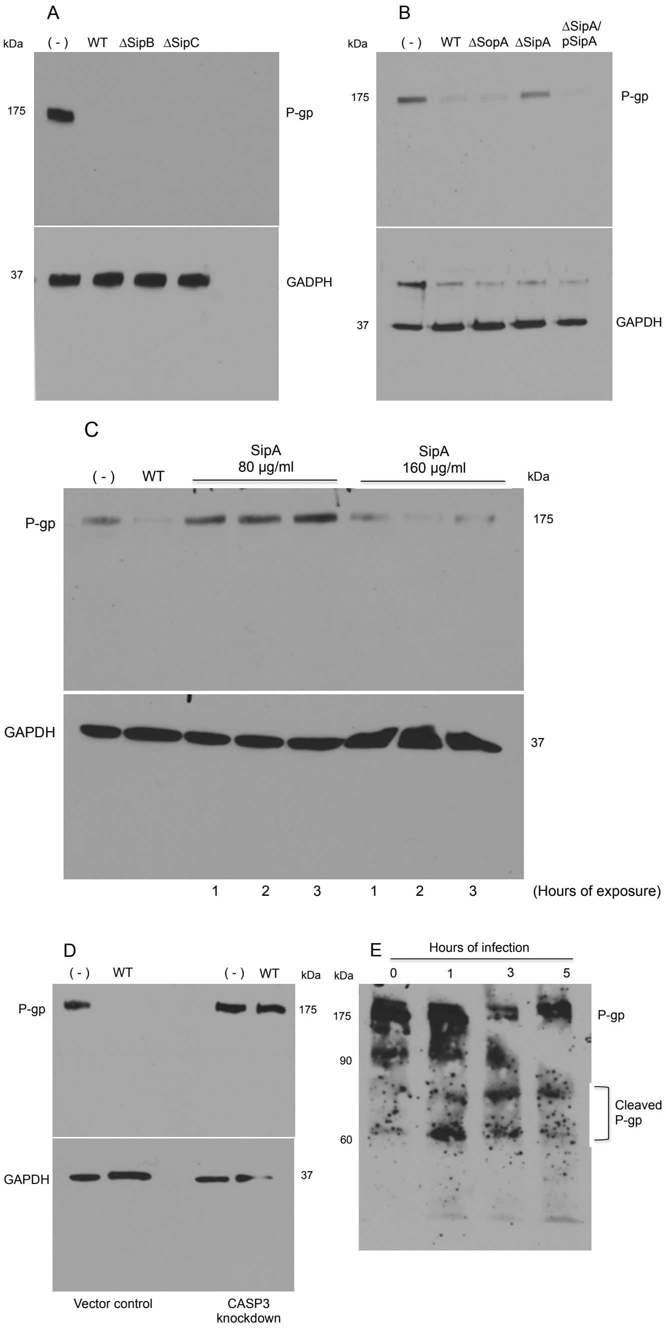

Supplementary Figure 17: Images of Uncropped Western blots.

(A) Figure 1A.

(B) Figure 1C.

(C) Figure 2A.

(D) Figure 3B.

(E) Figure 3D

Supplementary Reference

1. W. Haiss, N. T. Thanh, J. Aveyard, D. G. Fernig, Determination of size and concentration of gold nanoparticles from UV-vis spectra. *Analytical chemistry* **79**, 4215-4221 (2007).
